# Population Structure Plays a Key Role in Community Stability

**DOI:** 10.1111/ele.70272

**Published:** 2025-12-08

**Authors:** Àlex Giménez‐Romero, Christina Hernández, Meritxell Genovart, Roberto Salguero Gómez

**Affiliations:** ^1^ Department of Biology University of Oxford Oxford UK; ^2^ Instituto de Física Interdisciplinar y Sistemas Complejos (IFISC, CSIC‐UIB) Palma de Mallorca Spain; ^3^ Centre d'Estudis Avançats de Blanes (CEAB‐CSIC), Departament d'Ecologia i Complexitat Blanes, Girona Spain

## Abstract

The relationship between ecosystem complexity and stability remains unresolved and a mechanistic explanation for the stunning levels of biodiversity observed in communities and ecosystems is still lacking. The theoretical study of the stability of ecological communities has long been dominated by the assumption that populations are homogeneous. However, populations are structured, consisting of individuals that differ in multiple traits—such as size or developmental stage—with specific energetic demands and use of space and resources. Stage‐specific interactions, such as asymmetric competition for resources or predation targeting particular life stages, are widespread in nature and strongly shape ecological dynamics. Recent theoretical work further demonstrates that differences in juvenile versus adult foraging capacity and predation risk can promote the persistence of larger and more complex communities than those predicted by unstructured models. Here, we develop a general framework to integrate population structure into community stability analyses and show that stage‐dependent interactions are key to stability. Specifically, while cross‐stage predator–prey interactions enhance stability, competition across different stages destabilises the community. Our results offer new insights into the stability‐diversity paradox by showing that stage‐structured interactions can effectively increase the magnitude of negative feedbacks and compress the unstable region. Overall, we emphasise the critical role of population structure, an often neglected feature of natural systems, in the stability of ecological communities.

## Graphical abstract

Population structure is a widespread yet underappreciated feature of ecological communities. Here, we develop a general theoretical framework integrating life‐stage structure into community matrix models and show that stage‐specific interactions—especially asymmetric predator‐prey dynamics—can fundamentally alter stability outcomes. Our findings reveal that such cross‐stage interactions can stabilize communities previously deemed unstable, offering new resolution to the classic complexity–stability paradox.

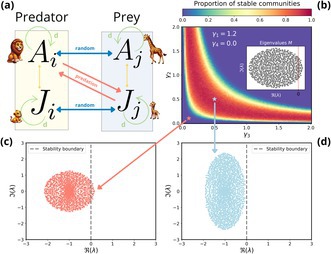

## 1 Introduction

The stability and diversity of ecological communities have been a central question in ecology for decades (Hutchinson 1961; Tilman 1999; Hautier et al. 2015; Donohue et al. 2016). Although most natural ecosystems are able to maintain their structure and function over time (Bai et al. 2004; Ives and Carpenter 2007), even in the presence of disturbances (Schindler 1990; Fischer et al. 2001), the mechanisms that promote this stability are not yet fully understood (Naeem et al. 1994). Ecologists have long argued that complexity promotes stability, allowing for a greater number of interactions between species that can buffer the system against disturbances (De Angelis 1975; McCann 2000). However, the idea of diversity enhancing stability was challenged over 50 years ago by the theoretical work of Robert May, who showed that large and diverse ecosystems are inherently unstable under randomly distributed interactions (May 1972). This apparent paradox sparked the so‐called stability‐diversity debate (De Angelis 1975; McCann 2000), prompting extensive research to uncover the mechanisms that promote stability in ecosystems (Ives and Carpenter 2007; Chesson 2000; Loreau and de Mazancourt 2013).

May's seminal work has greatly influenced the field of theoretical and applied ecology (May and McLean 2007; Landi et al. 2018), having inspired a large body of work on the stability of ecosystems (Pimm 1984; Allesina and Tang 2015). Over the past decades, a large body of work has demonstrated that community stability cannot be understood solely by species richness or connectance, but critically depends on the structure of interaction networks. The nature of species interactions is central: predator–prey dynamics promote stability, while mutualism and competition tend to destabilise communities (Allesina and Tang 2012), although mixed interaction types can yield stabilising effects under certain conditions (Mougi and Kondoh 2012; Qian and Akçay 2020). Likewise, the strength and distribution of interactions play a key role. Weak interactions can either stabilise or destabilise communities depending on context (Allesina and Tang 2012; Neutel et al. 2002), while omnivory (Pimm and Lawton 1978), consumer preferences and adaptive foraging (Kondoh 2003), and allometric scaling of predator–prey strengths (Brose et al. 2006) have all been shown to enhance robustness. At the network level, structural features such as nestedness and modularity (de Ruiter et al. 1995; Rooney et al. 2006; Thébault and Fontaine 2010; Grilli et al. 2016; Duan et al. 2023) strongly influence persistence and resilience. In addition, the explicit considerations that interactions among species are not always pair‐wise, but rather higher‐order (Grilli, Barabás, et al. 2017), and that the effect of the interactions is not always instantaneous but can occur with a delay (Pigani et al. 2022), have also been shown to build more robust and stable communities. Moreover, the more recent integration of transient dynamics (Hastings 2004; Hastings et al. 2018) via pseudospectral analyses (Tang and Allesina 2014; Caravelli and Staniczenko 2016) has shown that reactivity, an intermediate state between non‐reactivity and instability, can be used as an early‐warning signal for ecosystem collapse (Yang et al. 2023). Recent work has also shown that dispersal is another factor driving community (Baron and Galla 2020) or meta‐community stability (Nauta and De Domenico 2024). The most recent attempt to solve the complexity‐stability debate comes from considering that growth scales as a sublinear power law with biomass (Hatton et al. 2024). However, this idea has already met opposition from both theoretical and empirical studies. In sublinear‐growth models, coexistence emerges from the fact that per‐capita growth rates tend to infinity at low abundance, which is unrealistic (Aguadé‐Gorgorió et al. 2025), while microbial growth is never sublinear (Camacho‐Mateu et al. 2025). On the other hand, the consideration of non‐linear interactions seems a potentially valuable direction to solve this question (Arese Lucini et al. 2020; Mazzarisi and Smerlak 2024). Collectively, these findings demonstrate that ecological stability is deeply shaped by the structure of interactions. However, one key structural property has remained comparatively overlooked: the internal organisation of populations themselves. Population structure—arising from differences among juveniles, adults, or other stages—adds an additional layer of heterogeneity in species interactions, yet its role in community stability remains far less explored than other structural dimensions.

The theoretical study of the stability of ecological communities has long been dominated by the assumption that populations are homogeneous, in contrast to the heterogeneity typically acknowledged among species. However, populations consist of individuals that differ in multiple traits, such as age, size, and developmental stage, with specific energetic demands and use of space and resources (Vindenes and Langangen 2015). Such life‐cycle related variation—referred to as population structure—determines how individuals interact with one another and their environment throughout their life cycle. Moreover, these traits shape vital rates like survival, growth, and reproduction, which ultimately drive population dynamics. For instance, both in plants (Snell and King 1977; Bock et al. 2019; Salguero‐Gómez and Casper 2010) and animals (Minelli and Fusco 2010), reproduction and survival are often strongly size‐ or stage‐dependent. Insects provide a clear example, as individuals go through distinct developmental stages that may differ significantly in their size, diet, and, consequently, in vital rates such as survival or growth (Dennis et al. 1995). In many species, interspecific interactions can vary depending on the life cycle stage of the individuals involved. Asymmetric interspecific competition for resources between different life cycle stages (Hamrin and Persson 1986; Briggs et al. 2000; Byström and Andersson 2005; Cameron et al. 2007; Potter et al. 2019) or stage‐specific predation (Brooks and Dodson 1965; Pennington et al. 1986; Campbell 1991; Barber‐Meyer and Mech 2008; Zink and Rosenheim 2008; Mishra et al. 2012; Pote and Nielsen 2017; Giachetti et al. 2022), such as adult predators feeding preferentially on juvenile prey, has been thoroughly documented, highlighting the importance of considering population structure when assessing ecological dynamics.

Previous works have explored the effect of population structure on community dynamics (Roos et al. 2008; Rossberg and Farnsworth 2011; Rudolf and Rasmussen 2013; de Roos and Persson 2013; de Roos 2020a, 2020b, 2021) showing that the former can significantly influence ecological dynamics and stability of the latter. For example, asymmetries in the extent of food limitation between individuals in different life cycle stages can lead to overcompensation in stage‐specific density with increasing mortality, which can ultimately increase the overall population abundance (de Roos 2020b). Indeed, recent theoretical work has shown that differences in juvenile vs. adult foraging capacity and predation risk within populations result in larger and more complex communities than predicted by unstructured models (de Roos 2021). However, these insights are largely restricted to specific predator–prey models, leaving open the question of how population structure more generally—across different interaction types and in more complex community contexts—shapes ecological stability. In particular, it remains unclear whether the stabilising effects of stage‐specific asymmetries observed in food web models extend to other types of interactions, such as competition, mutualism, or mixed interaction networks. Addressing this gap requires a framework that can accommodate arbitrary numbers of life stages and diverse interaction types, enabling a systematic comparison of structured and unstructured models.

Here, we introduce a general framework to study the role of population structure in the stability of ecological communities. Unlike standard models, which assess interactions at the species level in an unstructured manner, we consider within‐population variation due to individual development during the life cycle. Our framework explicitly accounts for inter‐stage (e.g., adult–juvenile) and intra‐stage (e.g., adult–adult) interactions by taking into account the structures of the populations involved in the community interaction. This layered structure can accommodate any number of life stages for any number of species, and can then be used to systematically integrate this rich information into standard stability analyses. We examine the stability of the community under different types of stage‐symmetric and asymmetric interactions from a theoretical perspective, and we evaluate the relevance of our framework in a collection of empirical food web networks. Overall, we offer new insights into the stability‐diversity paradox by emphasising the potential contribution of population structure, an oftentimes neglected feature of natural systems. We finally propose a road map for future research, including extensions to further develop our framework.

The three main contributions of the paper are: (i) the derivation of the Structured Community Matrix, a generalisation of the traditional Community Matrix that accounts for the effect of population structure in community stability, (ii) the stability analysis across interaction types for an N‐species 2‐stage model under random matrix assumptions, and (iii) a robustness test of our results on 33 empirical food webs, which constrain the topology of the interaction networks and show that our results hold over a range of species richness and network connectivities.

## 2 Modelling Communities With Unstructured Populations

The stability of ecological communities is often tackled from a dynamical systems perspective (Van Meerbeek et al. 2021). Typically, in community ecology, a community is modelled as a set of interacting species in which the dynamics of their densities are described by a system of Ordinary Differential Equations (ODEs). Under this formalism, the local asymptotic stability of the system is studied with respect to a given feasible equilibrium, a fixed point of the dynamical system in which all species have positive population densities (Song et al. 2018). It turns out that the stability of the system is completely determined by the eigenvalues of the Jacobian matrix evaluated at the equilibrium, also known as the community matrix (Box 1). This approach enables the study of the stability of communities in a general and systematic way, and has been instrumental in the development of the field of theoretical ecology (Hastings et al. 2018; Schaffer 1985; Roberts 1987; Zhao 2003; May 2019). While the study of feasibility in ecological communities—examining whether all species maintain positive abundances at equilibrium—is an important and related problem (Song et al. 2018; Grilli, Adorisio, et al. 2017), our focus here is solely on stability.

Determining the exact form of the system of ODEs that describe the dynamics of a given species in a community can be challenging. The difficulty in doing so stems from the requirement for detailed knowledge of the interactions between species (Dieck Kattas et al. 2013). Indeed, field data on species interactions are limited by the vast number of potential interactions in diverse communities and the significant effort required to document them (Jordano 2016). Moreover, the variation in species interactions across space and time adds further complexity (Tang et al. 2023), making it difficult to distinguish between true negatives (i.e., where species do not interact) and false negatives (where interactions are occurring but undetected). Furthermore, empirical estimates of interaction strength are often biased, as they typically rely on species‐level summaries, losing critical detail about individual or contextual variations (Strydom et al. 2021). And even if these obstacles are overcome, it is still difficult to integrate the recorded interactions within a common framework (Pilosof et al. 2017). If this were not enough, even when interactions are observed, their quantitative representation is complicated by the choice of functional responses (e.g., Holling type I–III) (Holling 1965, 1966) and the form of growth density dependence, which remain central open questions in ecology (Hatton et al. 2024). Consequently, studying the stability of communities with a large number of species has remained a gargantuan task in Ecology.

BOX 1 **Dynamical Systems Perspective on Assessing Community Stability.**

Consider a community composed by S species in which the dynamics of the species densities, $N_{i}$, are described by a general system of ODEs which might depend on the population densities of all species in the community

(1) $$\frac{dN_{i}}{dt}=f_{i}\left(N_{1},\ldots ,N_{S}\right).$$

The function $f_{i}$ encodes the effects of the interactions among species, that is, how the density of a given species is affected by the density of another species. For instance, if the density of species i increases with an increasing density of species j, and the same happens the other way round, this interaction is regarded as mutualistic. On the contrary, if the density of two interacting species decreases as the density of the counterpart increases, the interaction is competitive.

Any equilibrium point of the system is given by the solution of

(2) $$\frac{dN_{i}}{dt}=f_{i}\left(N_{1},\ldots ,N_{S}\right)=0,$$

and is said to be feasible if all species have positive population densities (Song et al. 2018).

To study the stability of the system one can consider the dynamics of small perturbations of the species' densities, ξ, around a given feasible equilibrium. The dynamics of such perturbations are given by

(3) $$N\left(t\right)=N^{*}+\xi \left(t\right),$$

where $N^{*}$ is a vector of positive vector densities that is solution of Equation (2) (i.e., the feasible equilibrium) and ξ is the magnitude of the perturbation to the species' densities, considered small.

We obtain a linearised approximation of the system by taking a Taylor expansion of the system of ODEs around the equilibrium and discarding higher‐order terms:

(4) $$\frac{d\xi \left(t\right)}{dt}=M\xi ,$$

where M is the Jacobian matrix of the system evaluated at the equilibrium, also known as the community matrix, given by

(5) $$m_{\mathrm{ij}}={\left.\frac{\partial f_{i}}{\partial N_{j}}\right|}_{N^{*}},$$

where $m_{\mathrm{ij}}$ are the elements of the matrix M, that is, the linearisation of the ODEs at equilibrium densities, defined by the partial derivatives of the species growth rate with respect to the species density evaluated at the equilibrium.

Thus, the elements of the community matrix measure the effect of a small change in the density of species j on the growth rate of species i at a given feasible equilibrium.

The solution of the linearised system (4) is given by

(6) $$\xi_{i}\left(t\right)=\sum_{j=1}^{S}{ℭ}_{\mathrm{ij}}e^{\lambda_{j}t},$$

where ${ℭ}_{\mathrm{ij}}$ are constants that depend on the initial conditions and $\lambda_{j}$ are the eigenvalues of the community matrix M.

Thus, the stability of the system is determined by the sign of the real parts of the eigenvalues of the community matrix. If all eigenvalues have negative real parts, all terms in Equation (6) decay to zero and the system is locally asymptotically stable. However, if there exists even one eigenvalue with a positive real part, the corresponding term in Equation (6) grows exponentially, driving the population away from the equilibrium and rendering the system locally unstable.

In Section 1: Appendix S1 we show an example of the application of this framework to a simple 2‐species Lotka‐Volterra model.

Over half a century ago, May combined dynamical system's theory with the emergent theory of random matrices to develop a ground‐breaking framework to study the stability of arbitrarily ‘complex’ and large communities. This approach is based on the insight of modelling the community matrix as a large random matrix, thus overcoming the need to specify the details of the interactions between species by defining a specific system of ODEs from which to obtain the Jacobian (May 1972; Allesina and Tang 2015). In this way, general theoretical insights about community stability can be obtained. The model considers that species interact with some probability and then the sign (e.g., negative for competitive or positive for mutualistic) and strength of the interaction are randomly assigned. May's main result showed that a large ecosystem with random interactions is stable with full certainty (probability of stability = 1) whenever the inequality shown below holds.

(7) $$\sigma \sqrt{\mathrm{SC}}<1,$$

where σ is the standard deviation of the interaction strength between species in the community of interest, S is the number of species in the community, and C is the probability of interaction between species (Box 2). Thus large (i.e., S→∞) and/or complex (very interconnected C→1 or strongly interacting σ→∞) communities are rather likely to be unstable.

BOX 2 **: The Stability of Communities From a Random Matrix Approach.**

Consider a community composed by S species whose densities obey a given system of ODEs, so that its stability is determined by the eigenvalues of the community matrix M, that is, the Jacobian of the system of ODEs (Box 1). Because the elements of the community matrix, $m_{\mathrm{ij}}$, represent the effect of the density of species j on the growth rate of species i at the equilibrium point of the system, the off‐diagonal elements of the matrix (i≠j) correspond to interspecific interactions, while the diagonal elements (i=j) correspond to intraspecific interactions.

By specifying the sign relation between the off‐diagonal elements of the community matrix, the different nature of species interactions can be modelled. For instance, if species i and j have a mutualistic interaction, the density of species i increases with an increasing density of species j, and the same happens the other way round, so that the sign of both $m_{\mathrm{ij}}$ and $m_{\mathrm{ji}}$ is positive. In general, we can parametrize the following interaction types (Allesina and Tang 2012).

• **Competition:** $\mathrm{sign}\left(m_{\mathrm{ij}}\right)=\mathrm{sign}\left(m_{\mathrm{ji}}\right)=-$

• **Mutualism:** $\mathrm{sign}\left(m_{\mathrm{ij}}\right)=\mathrm{sign}\left(m_{\mathrm{ji}}\right)=+$

• **Predation/parasitism:** $\mathrm{sign}\left(m_{\mathrm{ij}}\right)=-\mathrm{sign}\left(m_{\mathrm{ji}}\right)$

• **Commensalism** $m_{\mathrm{ij}}=0,\mathrm{sign}\left(m_{\mathrm{ji}}\right)=+$

• **Amensalism** $m_{\mathrm{ij}}=0,\mathrm{sign}\left(m_{\mathrm{ji}}\right)=-$

On the other hand, it is often assumed that the species are self‐regulating (e.g., negatively density‐dependent), so that the diagonal elements of the community matrix are usually set to a negative value $m_{\mathrm{ii}}<0$. In addition, not all species necessarily interact with the rest, so species can be modelled to interact with others with given probability $C\in \left[0,1\right]$, and otherwise its interaction coefficient vanishes, $m_{\mathrm{ij}}=0$.

Overall, one can obtain the community matrix of randomly assembled communities by specifying the species' pair‐wise interaction probability (C), the magnitude of the diagonal coefficients (intraspecific interactions, d), and the sign and magnitude of the off‐diagonal coefficients (interspecific interactions, which are typically obtained from a given probability distribution). Then, using tools from random matrix theory, one can study the statistical properties of the eigenvalues of the community matrix and determine under which conditions the system is stable. For example, using the circular law, it can be deduced that the eigenvalue distribution of a S×S random matrix M with mean trace $E\left[\mathrm{Tr}\left(M\right)\right]=E\left[\sum_{i}m_{\mathrm{ii}}\right]=d$ and with independent and identically distributed entries of zero mean and variance σ, which are drawn with probability C, converges to a uniform distribution in a circle in the complex plane with centre $\left(d,0\right)$ and radius $\sigma \sqrt{\mathrm{SC}}$ as S→∞ (Allesina and Tang 2015; Tao et al. 2010). Importantly, this outcome is independent from the probability distribution from which the matrix elements are obtained.

This result is key to examine the stability of communities with random species interactions, such as the case studied by May (May 1972). In this case, the diagonal elements of the community matrix were fixed to $m_{\mathrm{ii}}=-1$ and the off‐diagonal elements were drawn from a Gaussian distribution of zero mean and variance σ, $N\left(0,\sigma \right)$, with probability C. Here σ can be understood as the interaction strength between species, and because any sign relation between the matrix coefficients can appear, any interaction type is modelled. Thus, the eigenvalues of the community matrix will be uniformly distributed in the complex plane following a circle of radius $\sigma \sqrt{\mathrm{SC}}$ centred at $\left(-1,0\right)$ (d=−1). Note that the abscissa of the complex plane corresponds to the real part of the eigenvalues of the community matrix, and the condition for the community to be stable is that all eigenvalues have negative real part (Box 1). Thus, the system will be stable when the eigenvalue distribution is completely contained in the left part of the complex plane with negative abscissa. We then conclude that whenever the centre of the circular eigenvalue distribution plus its radius is negative, $-1+\sigma \sqrt{\mathrm{SC}}<0$, the system will be stable with probability tending to one, recovering May's stability criterion (May 1972) (Equation (7))

(8) $$\sigma \sqrt{\mathrm{SC}}<1.$$

Indeed, the same result applies to the more general case in which the elements of the community matrix are given by any probability distribution with finite first and second moments and the mean of the diagonal elements is given by $E\left[m_{\mathrm{ii}}\right]=d$ (Allesina and Tang 2015). The stability criterion is given by

(9) $$\gamma =\sigma \sqrt{\mathrm{SC}}<d,$$

where $\gamma =\sigma \sqrt{\mathrm{SC}}$ is proportional to the interspecific interaction strength, as opposed to d that corresponds to the intraspecific interaction strength, that is, self‐regulation.

In addition, similar stability criteria can be obtained for specific types of interacting communities. For these cases, the derivation of the stability criteria implies the use of the elliptic law (Tao et al. 2010), a generalisation of the circular law for random matrices with correlated entries, which gives rise to the following criteria (Allesina and Tang 2012; Sommers et al. 1988):

(10) $$\begin{array}{ll} \text{Mutualistic}: & \gamma_{m}=\mathrm{\sigma C}\left(S-1\right)\sqrt{\frac{2}{\pi }}<d\\ \text{Competitive}: & \gamma_{c}=\sigma \left\{\sqrt{\mathrm{SC}}\left(1+\frac{2-2C}{\pi -2C}\right)\sqrt{\frac{\pi -2C}{\pi }}+C\sqrt{\frac{2}{\pi }}\right\}<d\\ \text{Predator}-\text{prey}: & \gamma_{p}=\frac{\pi -2}{\pi }\sigma \sqrt{\mathrm{SC}}<d \end{array}$$

In summary, a randomly assembled community will be locally asymptotically stable with probability approaching one when $\gamma \left(\sigma ,S,C\right)<d$, where S is the number of species in the community, C is the pair‐wise interaction probability between species, and σ is the strength of the interactions. The form of the function $\gamma \left(\sigma ,S,C\right)$ will depend on the interaction types present in the community (e.g., Equations 9 and 10). For a recent review on Lotka‐Volterra models and random matrix theory see (Allesina and Tang 2015; Akjouj et al. 2024).

While May's seminal work and the extensive research it inspired have significantly advanced our understanding of community stability, these studies typically rely on models that treat populations as homogeneous units. However, real populations are structured: individuals differ in age, size, or developmental stage, and these differences can fundamentally alter how species interact and respond to perturbations. To address this overlooked dimension, we develop a general framework that explicitly incorporates population structure into community stability analysis.

## 3 A General Framework to Address the Role of Population Structure in the Stability of Communities

Here, we introduce a generalised framework to examine the stability of communities with structured populations. We consider a community composed by S species and K life cycle stages for each species. These K stages could represent discrete developmental groups–such as juveniles and adults– or age‐ and size‐based groupings. What matters in this regard is that the groups represent biologically‐meaningful differences in the probability, direction, or strength of interactions among the species‐stage combinations.

Consider a dynamical system representing a community of age/stage‐structured populations. Generally, we may consider S species and K stages for each species. The dynamics of the system can be described by a general system of ODEs that describes the growth rate of each population stage as a function of the densities of all stages of all species (including the focal species) in the community,

(11) $$\frac{dN_{i}^{\left(k\right)}}{dt}=g_{i}^{\left(k\right)}\left(N_{1}^{\left(1\right)},\ldots ,N_{1}^{\left(K\right)},\ldots ,N_{S}^{\left(1\right)},\ldots ,N_{S}^{\left(K\right)}\right),$$

where $N_{i}^{\left(k\right)}$ denotes the population density of the stage k of species i, with i=1,…,S and k=1,…,K and the function $g_{i}^{\left(k\right)}$ encodes the effects of the interaction among the different stages of the different species in the community.

This set of ODEs can incorporate any desired ecological mechanisms (e.g., transfers between stages due to reproduction or ageing, any explicit functional form of species interaction, etc.). As explained in Box 1, the stability of the model is given by the real part of the largest eigenvalue of the Jacobian of the system of differential equations. However, we are interested in comparing the stability of the structured model in Equation (11), with K×S equations for the density of the different species' populations at each stage, to that of an equivalent unstructured model, which would have only S equations for the species' densities. In this way, the effect of structure on the stability of the community can be assessed.

To do so, we perform a change of variables. We can rearrange this system of ODEs by defining

(12) $$N_{i}=\sum_{k=1}^{K}N_{i}^{\left(k\right)}$$

(13) $$Z_{i}^{\left(k\right)}=\frac{N_{i}^{\left(k\right)}}{N_{i}}\;\forall k=1,\ldots ,K-1,$$

where $N_{i}$ represents the total density of species i and $Z_{i}^{\left(k\right)}$ is the fraction of population density of species i at stage k. The fraction of population density of species i at stage k=K is automatically defined by

(14) $$Z_{i}^{\left(K\right)}=\left(1-\sum_{k=1}^{K-1}Z_{i}^{\left(k\right)}\right)$$

We can then express our system of ODEs Equation (11) as a function of total species' densities Equation (12) and fraction of stages Equation (13),
(15)

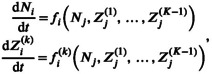

where k∈1,…,K−1 and j∈1,…,S.

Our initial system of ODEs, Equation (11), has S×K equations that describe the dynamics of the densities of each of the stages for each of the species in the community. After the change of variables, the new system of ODE's, Equation (15), describes the dynamics of the total density of each of the species, Equation (12), and the fraction of population density at each of the stages for each of the species, Equation (14).

We define the structured community matrix, $M_{S}$, as the Jacobian of the age/stage structured population model after the change of variables, Equation (15), evaluated at a feasible equilibrium point (denoted by *), which is a SK×SK matrix given by

(16) $$M_{S}={\left(\begin{array}{cccc} \frac{\partial f_{i}}{\partial N_{j}} & \frac{\partial f_{i}}{\partial Z_{j}^{\left(1\right)}} & \ldots & \frac{\partial f_{i}}{\partial Z_{j}^{\left(K-1\right)}}\\ \frac{\partial f_{i}^{\left(1\right)}}{\partial N_{j}} & \frac{\partial f_{i}^{\left(1\right)}}{\partial Z_{j}^{\left(1\right)}} & \ldots & \frac{\partial f_{i}^{\left(1\right)}}{\partial Z_{j}^{\left(K-1\right)}}\\ \vdots & & & \vdots \\ \frac{\partial f_{i}^{\left(K-1\right)}}{\partial N_{j}} & \frac{\partial f_{i}^{\left(K-1\right)}}{\partial Z_{j}^{\left(1\right)}} & \ldots & \frac{\partial f_{i}^{\left(K-1\right)}}{\partial Z_{j}^{\left(K-1\right)}} \end{array}\right)}^{*}=\left(\begin{array}{cccc} M & M_{S}^{12} & \ldots & M_{S}^{1K}\\ M_{S}^{21} & M_{S}^{22} & \ldots & M_{S}^{2K}\\ \vdots & & & \vdots \\ M_{S}^{K1} & M_{S}^{K2} & \ldots & M_{S}^{\mathrm{KK}} \end{array}\right)$$

in which each element of the depicted matrix is, in turn, a S×S matrix and the top‐left block, the sub‐matrix denoted as M, is simply the traditional community matrix. In Section 2: Appendix S1 we provide a simpler example by deriving the structured community matrix for a general S‐species two‐stage model.

The change of variables that we performed allows us to express the upper left sub‐matrix of the Jacobian, M, as the traditional community matrix, that is, the elements of this sub‐matrix are ${\left(\frac{\partial f_{i}}{\partial N_{j}}\right)}^{*}$. In this way, we can directly compare the eigenvalues of the traditional community matrix, that does not take into account stage structure, to those of our full stage‐structured matrix. In turn, this comparison allows us to understand the effect of population structure on the stability of the community. The remaining sub‐matrices in the first row represent the effects of the perturbation in the fractional density of the different stages of the species on the total species' densities, while the remaining sub‐matrices of the first column represent the effects of the perturbation in the total species' densities on the fractional density of the different stages of the species. The rest of the sub‐matrices represent the effect of the perturbation in the fractional density of the different stages of the species on the fractional density of the different stages of the species. For instance, the sub‐matrix $M_{S}^{34}$ represents the effect of the perturbation in the fractional density of stage 4 of any species on the fractional density of stage 3 of any other species, including itself (recall that each sub‐matrix is a S×S matrix).

The stability of the system is determined by the eigenvalues of the structured community matrix, $M_{S}$, which generalises the community matrix to structured populations. Our framework can also accommodate species with differing number of life cycle stages. To do so, one only needs to develop the model for $K_{\max}$ stages (where $K_{\max}$ denotes the maximum number of life cycle stages of any species' population in the community) and set to 0 the interaction coefficient of the non‐existing stages of populations with a number of stages smaller than $K_{\max}$.

The approach of random matrices can be applied to the structured community matrix, allowing the study of the stability of ecological systems with structured populations in a general and systematic way. However, obtaining a general analytical solution for the stability criterion of communities with structured populations is arduous. In the following, we present some examples of how population structure can influence the stability of communities, showing that one can obtain opposite results when considering unstructured models.

## 4 Cross‐Stage Predator–Prey Interactions Stabilise Communities

To demonstrate the applicability of our approach, we first extend the insights of de Roos (de Roos 2021) to a broader context. Using a specific system of differential equations, de Roos highlighted that the differential foraging abilities and predation risks between juvenile and adult individuals within populations can enhance community stability. In this example, we parametrise our general model to represent a community featuring adult–juvenile predator–prey interactions, while adult–adult and juvenile–juvenile interactions are treated as random, allowing for any sign in their interaction coefficients (i.e., any type of interaction). In this particular case, the structured community matrix is a simpler 2S×2S matrix, which can be expressed as the following 2×2 block matrix (of S×S sub‐matrices),

(17) $$M_{S}=\left(\begin{array}{cc} M & M_{S}^{12}\\ M_{S}^{21} & M_{S}^{22} \end{array}\right)=\left(\begin{array}{cc} M_{1} & M_{2}\\ M_{3} & M_{4} \end{array}\right),$$

where we have simplified notation in the second step of Equation (17) to make the example easier to follow.

$M_{1}=M$ is the traditional community matrix, and it represents the pair‐wise interactions between the species' populations within the community, where the interaction does not take into consideration the structure of either species' population. $M_{2}$ represents the interactions between the total population of one species and the fraction of juveniles of another. $M_{3}$ represents the interactions between the fraction of juveniles of one species and the total population of another. Finally, $M_{4}$ represents the interactions between the fractions of juveniles in pair‐wise combinations of species in the community.

To demonstrate the impact of population structure on community stability, we explored how interactions between different life cycle stages influence the overall dynamics. We first considered random interactions in $M_{1}$ and $M_{4}$, and predator–prey interactions in $M_{2}$ and $M_{3}$. As such, the diagonal elements of $M_{1}$ and $M_{4}$ were set to −d, corresponding to density‐dependent regulation in each stage, while their off‐diagonal elements were drawn from normal distributions with mean zero and variance $\sigma_{1}$ and $\sigma_{4}$, respectively. In contrast, the elements of $M_{2}$ and $M_{3}$ were obtained by considering the effect of cross‐stage predator–prey interactions. Mathematically, the matrices $M_{1}$ and $M_{4}$ were parametrised as,

(18) $$M_{1}=\left\{\begin{array}{lll} m_{\mathrm{ij}}=-d & \text{if} & i=j\\ m_{\mathrm{ij}}=N\left(0,\sigma_{1}\right) & \text{if} & i\neq j \end{array}\right.,\;M_{4}=\left\{\begin{array}{lll} m_{\mathrm{ij}}=-d & \text{if} & i=j\\ m_{\mathrm{ij}}=N\left(0,\sigma_{4}\right) & \text{if} & i\neq j \end{array}\right..$$

while the matrices $M_{2}$ and $M_{3}$ were parametrised as,

(19) $$M_{2}=\left\{\begin{array}{l} m_{\mathrm{ij}}=|N\left(0,\sigma_{2}\right)|,m_{\mathrm{ji}}=-|N\left(0,\sigma_{2}\right)|\;\text{if}\;i\;\text{predates}\;j\\ m_{\mathrm{ij}}=-|N\left(0,\sigma_{2}\right)|,m_{\mathrm{ji}}=|N\left(0,\sigma_{2}\right)|\;\text{if}\;j\;\text{predates}\;i \end{array}\right.$$

(20) $$M_{3}=\left\{\begin{array}{l} m_{\mathrm{ij}}=-|N\left(0,\sigma_{3}\right)|,m_{\mathrm{ji}}=|N\left(0,\sigma_{3}\right)|\;\text{if}\;i\;\text{predates}\;j\\ m_{\mathrm{ij}}=|N\left(0,\sigma_{3}\right)|,m_{\mathrm{ji}}=-|N\left(0,\sigma_{3}\right)|\;\text{if}\;j\;\text{predates}\;i \end{array}\right.$$

where $N\left(\mu ,\sigma \right)$ denotes a Gaussian distribution with mean μ and variance σ. To parametrize nonrandom interaction types, we use the absolute value of normally distributed coefficients, following standard practice (Allesina and Tang 2012), which results in a folded normal distribution. Importantly, by assigning different σ values to the various sub‐matrices, we can modulate the strength of interactions between life stages, including fully deactivating specific interaction types when σ=0.

We generated an ensemble of 1000 random communities with stage‐structured populations by drawing the interaction coefficients of the structured community matrix, $M_{S}$, from normal distributions, following Equations ((18), (19), (20)). A community was classified as stable if all the eigenvalues of the structured community matrix had negative real parts. Otherwise it was denoted as unstable. The proportion of stable communities shown in the figures therefore corresponds to the fraction of simulated communities within the ensemble that satisfy this stability criterion. We provide the code used to perform these simulations, along with some examples, in a GitHub (Giménez‐Romero n.d.) and a Zenodo (Giménez‐Romero n.d.) repository.

Each of the sub‐matrices is parametrised by $\sigma_{i}$, the interaction strength between life stages (Equations (18), (19), (20)), but because S=200 and C=1 are fixed parameters in our simulations, $\gamma_{i}$ is proportional to $\sigma_{i}$. This provides a convenient scaling, as the interaction matrices can also be parametrised in terms of $\gamma_{i}$ (Equation (10)): $\gamma_{1}=\sigma_{1}\sqrt{\mathrm{SC}}$, $\gamma_{2}=\frac{\pi -2}{\pi }\sigma_{2}\sqrt{\mathrm{SC}}$, $\gamma_{3}=\frac{\pi -2}{\pi }\sigma_{3}\sqrt{\mathrm{SC}}$ and $\gamma_{4}=\sigma_{4}\sqrt{\mathrm{SC}}$. Thus, $\gamma_{1}$ is proportional to the interaction strength between total population densities, $\gamma_{2}$ and $\gamma_{3}$ to the cross‐stage interaction strengths, between total population densities and fractions of juveniles, and $\gamma_{4}$ to the interaction strength between fractions of juveniles of different species. For simplicity, we set $\sigma_{4}=0\Rightarrow \gamma_{4}=0$, this is, we consider no juvenile‐juvenile interactions in the community. Detailed information on the parametrisation of the structured community matrix is provided in Section 4: Appendix S1.

**FIGURE 1:**
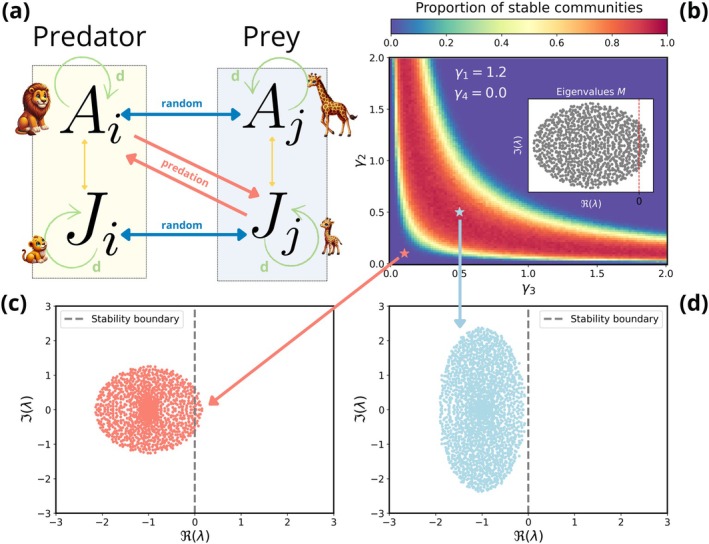
Stability analysis of a community composed of structured populations (adults and juveniles) with cross‐stage predator–prey interactions. (a) Schematic representation of the parametrised community, where $A_{i}$, $A_{j}$, represent the density of adults of two example species of the community and $J_{i}$, $J_{j}$ represent the corresponding density of juveniles. Adult–adult and juvenile‐juvenile interactions between species are set randomly, so any interaction type can occur, while adult–juvenile interactions between species are predator–prey. After the parametrisation of the structured community matrix, $M_{S}$, $\gamma_{1}$ represents the interaction strength between total population densities, $\gamma_{2}$ and $\gamma_{3}$ represent the cross‐stage interactions strengths between total population densities and fraction of juveniles, and $\gamma_{4}$ represents the interaction strength between fraction of juveniles. (b) Proportion of stable communities as a function of the cross‐stage interaction strengths, $\gamma_{2}$ and $\gamma_{3}$, based on an ensemble average of $10^{3}$ simulations with communities of S=200 different species and all species interact (C=1). The total population to total population interaction strength is set to $\gamma_{1}=1.2$, which is higher than the self‐regulation term, d=1. The interaction strength between fraction of juveniles is set to $\gamma_{4}=0$, considering for simplicity that juveniles do not interact with other juveniles. The traditional community matrix that considers unstructured populations ($\gamma_{1}=1.2$) has positive eigenvalues (inset) so that the community would be labelled as unstable. However, there is a region of stability. (c, d) Complex plane displaying the distribution of the real and imaginary part of the eigenvalues of the structured community matrix, $M_{S}$, where the abscissa and the ordinate are the real and imaginary parts of the eigenvalues, respectively. The dashed grey line indicates $ℜ\left(\lambda \right)=0$, that is, the onset of instability. (c) Eigenvalues of the structured community matrix for $\gamma_{2}=\gamma_{3}=0.1$. The cross‐stage interaction strength is not enough to provide stability to the community. (d) Eigenvalues of the structured community matrix for $\gamma_{2}=\gamma_{3}=0.5$. In this case, the cross‐stage interaction strength is enough to provide stability to the community.

The key consideration is that we set $\gamma_{1}>d$ (i.e., the interaction strength among the total population densities exceeds the self‐regulation capacity of each population), and as such $M_{1}=M$ has at least one positive eigenvalue. If we only consider the interactions among the total population densities in the community (the sub‐matrix $M_{1}$ in Equation (17)), we would conclude that the community is unstable. However, when explicitly considering population structure, the stability of the community depends on the cross‐stage interaction strength, $\gamma_{2}$ and $\gamma_{3}$ (Figure 1). We reach the same conclusion when the adult–adult interactions are set to be competitive or mutualistic (Figure S2), as well as when interaction strengths are drawn from a truncated normal distribution instead of a folded normal distribution (Figure S4).

## 5 Asymmetry Between Intra‐ and Inter‐Stage Interaction Types Is Key to Community Stability

To demonstrate that our framework is general and flexible, we then extended our analyses by setting different types of interactions among population stages within or across species in a community. To illustrate its utility while keeping clarity, we analyse the simplest scenario: communities comprising populations structured into two stages. As in the previous model, for simplicity, we refer to these stages as juvenile and adult stages, but they could be any other traits that structure the population.

This setup allows us to systematically explore the effects of stage‐specific interactions, such as mutualistic (+/+), competitive (−/−), or trophic (e.g., predator–prey) relationships, on overall community stability. For instance, we investigate cases where adult–adult interactions are competitive while adult–juvenile interactions are predatory, cases where adult–adult interactions are mutualistic while adults compete with juveniles, and many other variations, demonstrating the versatility of the framework in analysing diverse interaction configurations. Detailed information on the parametrisation of the structured community matrix for each case is provided in Sections 3 and 4: Appendix S1.

**FIGURE 2:**
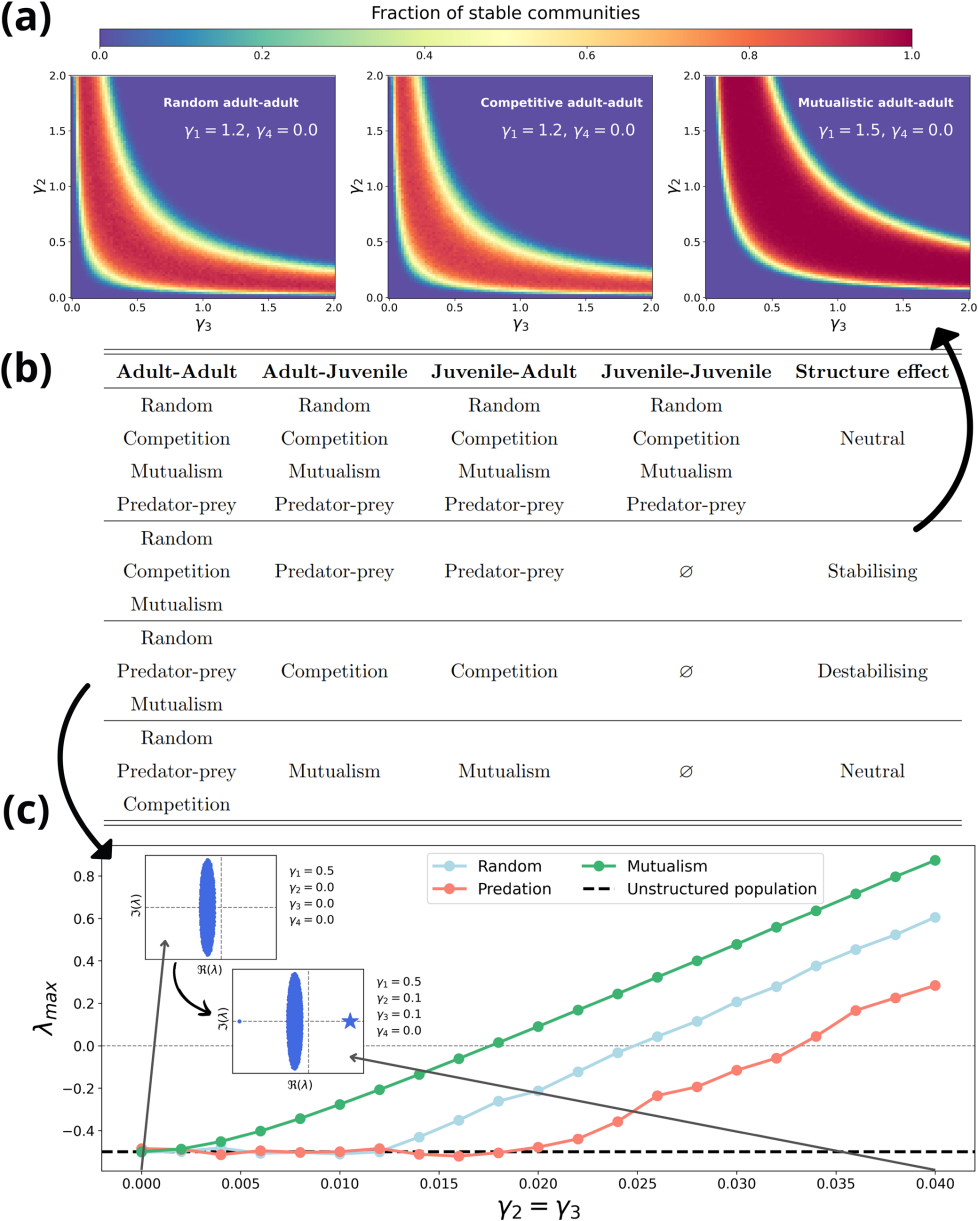
Effect of adding population structure on the stability of communities. (a) Stabilising effect of structured predator–prey interactions regardless of the adult–adult interaction type. (b) Comparison of the stability of communities with no population structure (each species is represented only once) to the stability of communities with a juvenile and adult class for each species. We refer to the effect as stabilising when the region of the parameter space where the community is stable is larger for the structured than the unstructured model, as occurs in panel (a). We refer to the effect as destabilising when the community with structured populations is unstable in regions of the parameter space where would be stable in the unstructured case, as happens in panel (c). The effect is neutral when the region of stable communities is the same for the structured and unstructured models. Each row of the table corresponds to a given parametrization of the structured community matrix, with the interaction type between the different life stages of the species in the community described by the columns. (c) Destabilising effect of structured competitive interactions, regardless of the adult–adult interaction type. When there are cross‐stage interactions, $\gamma_{2}=\gamma_{3}\neq 0$, the community becomes unstable, that is, the largest eigenvalue of the structured community matrix, $\lambda_{\max}$ (blue star), becomes positive for very small values of $\gamma_{2}=\gamma_{3}$. The corresponding unstructured model would be stable (dotted black line) as the total population to total population interaction strength is $\gamma_{1}=0.5<1$, and as shown at the top inset there are no positive eigenvalues in the community matrix.

In Figure 2, we show the comparison of the stability outcomes of structured and unstructured communities, highlighting how distinguishing life stages can reshape stability predictions. One key finding is that when inter‐ and intra‐stage interactions are of the same type (e.g., all competitive), introducing population structure has little to no effect on community stability (Figure 2 and Figure S1). However, when inter‐ and intra‐stage interaction types differ, novel stabilising or destabilising mechanisms emerge. For example, as previously shown, we recover the results of de Roos (de Roos 2021) by incorporating predator–prey interactions between adults and juveniles of different species (Figure 1). In fact, we show that these inter‐stage predator–prey interactions stabilise the community, regardless of the nature of intra‐stage interactions (Figure 2 and Figure S2). In addition, we show that the result is robust to different values of the pair‐wise interaction probability C (Figure S3).

Conversely, we show that competition across life stages consistently promotes instability (Figure 2 and Figure S5). Interestingly, our results show that mutualistic cross‐stage interactions have minimal influence on community stability compared to unstructured models (Figure 2 and Figure S6). This result suggests that the stabilising effects of population structure depend critically on the asymmetry and regulatory dynamics introduced by specific interaction types.

## 6 Cross‐Stage Predation Provides Stability to Empirical Food Webs

To evaluate the relevance of our framework in real‐world settings, we applied it to a collection of empirical food web networks under the assumption that predator–prey interactions occur between different life cycle stages of the interacting species. Unfortunately, empirical data explicitly documenting interaction strengths at the level of life cycle stages is currently lacking, but species‐level food web data still provide a biologically meaningful constraint on the underlying network structure. This approach allows us to explore the consequences of introducing structured interactions in ecologically realistic topologies.

We analysed 33 food webs obtained from the Web of Life database. These food webs constrained the number of species, S, connectivity, C, and the structural properties of the interaction networks empirically. In particular, the analysed food webs consisted of a number of species that spanned from S=14 to S=249 and connectivity values from C=0.035 to C=0.485 (Figure S7). For each food web, we assumed that every species is composed of two stages—juveniles and adults—and structured the interaction matrix accordingly. Adult–adult and juvenile–adult interactions were treated as random, while adult–juvenile interactions followed a predator–prey structure consistent with the species‐level link direction in the original food web. Juvenile‐juvenile interactions were omitted for simplicity. For details about the parametrisation of the structured community matrix see Section 5: Appendix S1.

**FIGURE 3:**
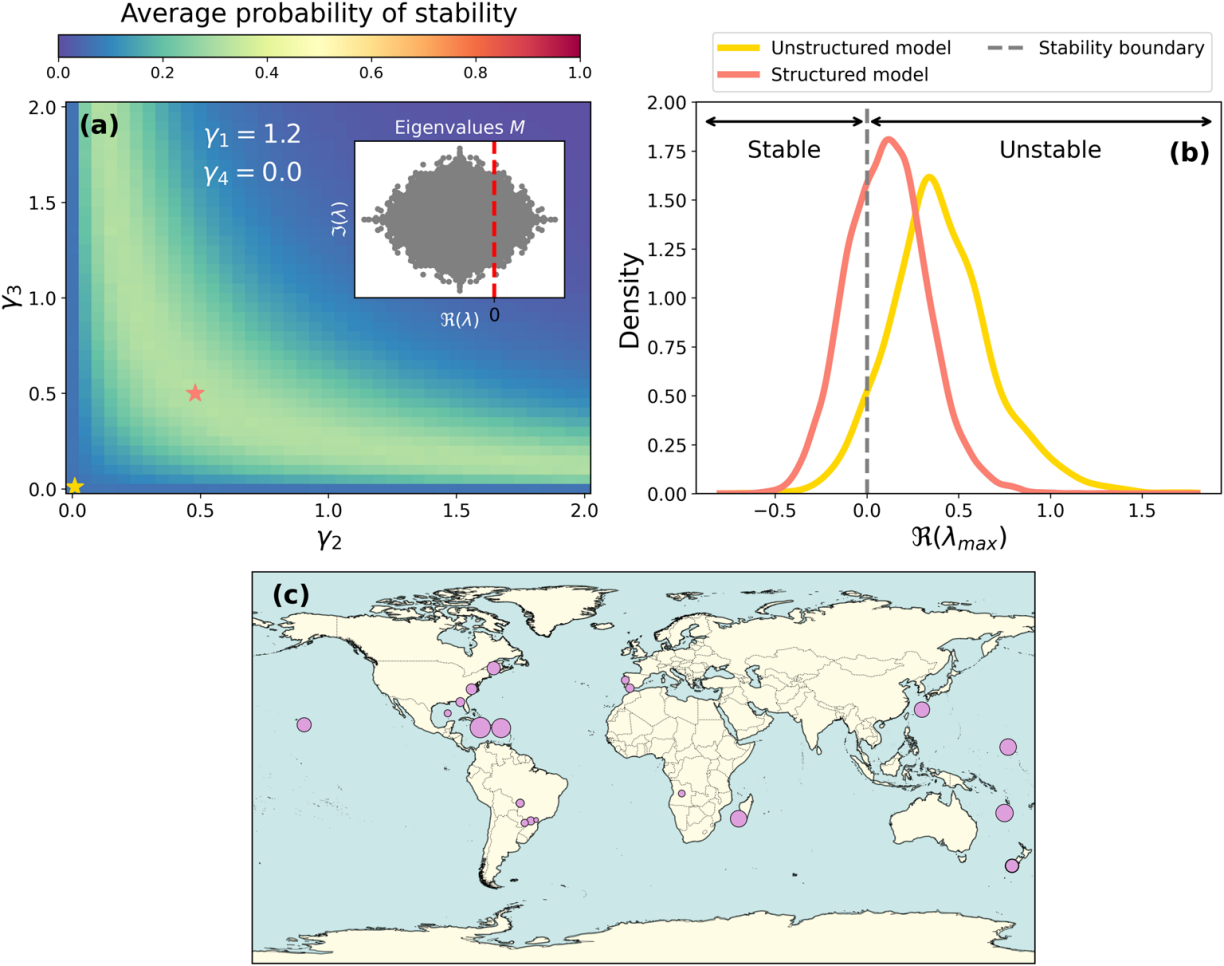
Stability of empirical food webs with cross‐stage predation. (a) Average stability of 33 empirical food webs with adult–juvenile predator–prey interactions as function of the cross‐stage interaction strength, $\gamma_{2}$ and $\gamma_{3}$. The inset shows the eigenvalue distribution of the corresponding unstructured population model for each of the communities, which is always unstable as $\gamma_{1}>d=1$ (b) Distribution of the maximum eigenvalue of the empirical networks for $\gamma_{2}=\gamma_{3}=0$ (yellow line, yellow star), equivalent to an unstructured model, and for $\gamma_{2}=\gamma_{3}=0.5$ (red line, red star). (c) Geographic location of the different food webs considered. The size of the dots are proportional to the number of species in the food web.

We parametrised each structured community matrix as in our theoretical analysis, with intraspecific regulation set to d=1 and the adult–adult interaction strength fixed at $\gamma_{1}=1.2$, ensuring that the corresponding unstructured community matrix would be unstable. We then examined how varying the strengths of cross‐stage predator–prey interactions ($\gamma_{2}$ and $\gamma_{3}$) affected stability. As shown in Figures S8 and S9, most empirical networks that were unstable under an unstructured formulation became stable when structured predator–prey interactions between life cycle stages were explicitly introduced. This trend is summarised in Figure 3a, which shows the average probability of stability across all 33 food webs as a function of the cross‐stage interaction strengths, $\gamma_{2}$ and $\gamma_{3}$. Indeed, for intermediate cross‐stage interaction strengths ($\gamma_{2}=\gamma_{3}=0.5$), the distribution of Reλmax is pushed to the left (i.e., towards the stable region, Reλmax<0) in comparison to the distribution of the largest eigenvalue of the corresponding unstructured model (Figure 3b). In Figure 3c, we show the geographic location of the analysed empirical food webs, with dot size proportional to the number of species in the food web.

These results demonstrate that stage‐structured interactions, particularly asymmetric predator–prey links across life stages, can have a strong stabilising effect even in empirically grounded systems.

## 7 Discussion

Our framework provides a general approach to studying the stability of ecological communities with structured populations, extending current approaches from dynamical systems and random matrix theory (May 1972; Allesina and Tang 2015, 2012; de Roos 2021). Unlike frequently used approaches that treat populations as homogeneous entities (Oksanen et al. 2013; Novak et al. 2016), our framework integrates heterogeneity to study how age, size, behavioural or other individual traits may modulate community responses to environmental fluctuations. By explicitly integrating biologically meaningful differences across life cycle stages into stability analyses, our framework extends traditional community matrix models to address the complexities inherent in natural ecosystems (de Roos 2020a, 2020b). The framework allows for the systematic examination of different types of cross‐stage interactions, while remaining flexible enough to accommodate diverse life‐history traits, varying numbers of life stages per species, and interaction patterns. As such, our approach is widely applicable to a broad range of ecological contexts.

Population structure introduces mechanisms that can drastically influence stability and resilience (Miller and Rudolf 2011). Our theoretical results, both for randomly assembled communities and empirical food webs, suggest that structures of interacting populations significantly reshape the boundaries of stability, offering new insights into the interplay of cross‐life cycle stage dynamics and network complexity. In particular, we found that predator–prey relationships between life cycle stages can enhance the stability of ecological communities. This type of asymmetry is not only a theoretical construct, but is widespread in natural systems. Life cycle stage‐specific predation has been observed in freshwater (Brooks and Dodson 1965; Campbell 1991), marine (Pennington et al. 1986; Zink and Rosenheim 2008; Giachetti et al. 2022) and terrestrial ecosystems (Barber‐Meyer and Mech 2008; Mishra et al. 2012; Pote and Nielsen 2017) and, in turn, across a wide range of taxa. In freshwater systems, classical studies have demonstrated that alewives preferentially feed on larger zooplankton such as *Daphnia* and *Diaptomus*, but both in their later developmental stages (Brooks and Dodson 1965), and that both vertebrate and invertebrate planktivores exhibit strong size and life cycle stage selectivity in their prey choices (Campbell 1991). In marine systems, zooplanktonic predators, including crustaceans and hydromedusae, exhibit differential feeding rates on embryonic and larval stages of echinoderms (Pennington et al. 1986), while ascidian species are most vulnerable to predation during early post‐settlement stages in benthic habitats (Giachetti et al. 2022). Terrestrial studies further support the ubiquity of stage‐structured predator–prey interactions. Adults of big‐eyed bugs (*Geocoris* spp.) suppress early instars of the western tarnished plant bug ( *Lygus hesperus* ) but not later developmental stages, indicating a strong ontogenetic asymmetry in predation pressure within insect communities (Zink and Rosenheim 2008). Similarly, ontogenetic changes in predation and conversion efficiency in four coccinellid beetle species have been reported, with early instars and young adults being the most efficient consumers of aphids (Mishra et al. 2012). Collectively, these findings suggest that life cycle stage‐asymmetric predation, particularly where adult individuals target juvenile stages of other species, is a pervasive feature of ecological communities and likely plays a critical but underappreciated role in shaping community stability. This observation aligns with the stabilising patterns predicted by our framework, reinforcing the ecological plausibility of the results.

The main limitation of using our framework together with random matrices precisely lies in the parametrisation of the structured community matrix. The effect of a change in density of the total population of one species on the fractional density of a particular stage of another is not straightforward to derive even when assuming a given interaction type between the species. For instance, we have considered that competitive adult–adult interactions have a negative impact on the total density of the species while adult–juvenile competition has a negative impact on the fractional density of juveniles. The rationale is that competitive pressure in adults can decrease fecundity or adult density (decreasing total species density) while competitive pressure in juveniles decreases both adult and juvenile density (so that the fractional density of juveniles relative to the total population also decreases). However, there may be other ways to characterise competitive pressure on adults and juveniles. Similarly, stage‐structured interactions might display an inherent structure in the Jacobian matrix (de Roos 2021), which is not accounted for when using random matrices. In addition, our findings so far have mostly dealt with randomly assembled communities and have not attempted to study the effect of the underlying interaction network structure. In natural systems, species interactions are not random; rather they are shaped by long‐term co‐adaptation and evolutionary pressures that influence both the types and strengths of interspecies relationships (Weber et al. 2017). This structured interaction network, often characterised by specific patterns like nestedness (Duan et al. 2023) or modularity (Grilli et al. 2016), likely interacts with population structure in ways that significantly influence community stability and resilience. While our empirical application incorporates real interaction topologies—supporting the ecological plausibility of our theoretical insights—we have not yet investigated in detail how specific network properties interact with population structure to affect community stability. Finally, feasibility constraints might modulate the size of the stable region,

These limitations can be addressed with future research. The effect of different inter‐stage interaction types (e.g., competitive adult–adult interactions) on the total population or fractional density of the stages can be analytically determined by considering specific functional forms for stage‐structured population dynamics. The challenge lies in mapping the effect of different interaction types on the density of the stages to the effect on the density of the total population and fraction of stages. In addition, the nuance of ecological communities could be investigated in our framework by considering different network‐generating mechanisms that further constrain the elements of the structured community matrix. Other future directions include incorporating additional dimensions of population heterogeneity, such as sex or behaviour, that may also influence interactions within and between species (Gissi et al. 2024) or extending the analysis to address, feasibility constraints (Song et al. 2018) or transient dynamics (Hastings et al. 2018) using pseudospectra (Gupta et al. 2025). We argue that these proposed future extensions will provide enhanced realism to the complex picture of community behaviour, allowing for the exploration of both long‐term stability and transient dynamics, a critical feature in understanding ecosystems under perturbations (Capdevila et al. 2020). Finally, validating structured community models with empirical data across diverse ecosystems remains paramount. A first step could be to investigate the typical patterns of inter‐ and intra‐stage interactions in natural communities that favour stability. These efforts will prove key in advancing predictive ecological modelling and informing conservation strategies that support biodiversity and ecosystem function.

## Author Contributions

R.S.G. conceptualised the project with key inputs from A.G.‐R. A.G.‐R., C.H., M.G. and R.S.G. and conducted investigations. A.G.‐R. performed the mathematical development, numerical analyses and simulations. A.G.‐R. wrote the first draft of the manuscript with key inputs and guidance from R.S.G. All authors contributed substantially to the final version of the manuscript and revisions.

## Funding

This work was funded by a NERC Pushing the Frontiers grant (NE/X013766/1) to R.S.G. A.G.‐R. was supported by grants CYCLE (PID2021‐123723OB‐C22), funded by MICIU/AEI/10.13039/501100011033 and by ERDF, EU; by grant PID2021‐124731NB‐I00 and by IMOVE (IMOVE24042) from the Spanish National Research Council (CSIC) and hosted by R.S.G. during this work. The work was also partially supported by the Spanish Ministerio de Ciencia e Innovación/AEI and EU‐FEDER funds (PID2021‐124731NB‐I00, PIE202230I133). C.H. was additionally supported by a Marie Curie Fellowship [MSCA DensPopDy #10115386] with funding through UKRI [grant number EP/Z002826/1], hosted by R.S.G.

## Supporting information


**Appendix S1:** ele70272‐sup‐0001‐supinfo.pdf.

**Data S1:** ele70272‐sup‐0002‐DataS1.png.

## Data Availability

The food webs used in this study are publicly accessible at Web of Life (Web of Life n.d.). The code used to perform the numerical analyses and simulations is publicly accessible in a GitHub (Giménez‐Romero et al. n.d.) and a Zenodo repository (Giménez‐Romero et al. n.d.).
