# Supplementary material for: Population Structure Plays a Key Role in Community Stability
Source: Ecol Lett. 2025 Dec 8;28(12):e70272. doi: 10.1111/ele.70272 (PMC12685569; doi:10.1111/ele.70272)
Supplement: Supplementary file 1 — Appendix S1: ele70272‐sup‐0001‐supinfo.pdf. [file ELE-28-0-s001.pdf]

Supplementary Information for

Population structure plays a key role in community stability

Àlex Giménez Romero*[1,2,3], Christina Hernández[1], Meritxell Genovart[3], and Roberto Salguero-Gómez†[2]

[1] *Instituto de Física Interdisciplinar y Sistemas Complejos (IFISC, CSIC-UIB), Campus UIB, 07122 Palma de Mallorca, Spain*
[2] *University of Oxford, Department of Biology, South Parks Road, Oxford OX1 3RB, UK*
[3] *Centre d'Estudis Avançats de Blanes (CEAB-CSIC), Departament d'Ecologia i Complexitat, Blanes Girona, Spain*

October 29, 2025

1 Example of the derivation of the community matrix for a two-species Lotka-Volterra model

Here we provide an example of how the framework explained in Box 1 on the main text applies to a two-species Lotka-Volterra model.

In Box 1, we consider a community composed by S species in which the dynamics of the species densities, N_i, are described by a general system of ODEs which might depend on the population densities of all species in the community

$$\frac{\mathrm{d}N_i}{\mathrm{d}t} = f_i(N_1, \ldots, N_S) \ . \tag{1}$$

For a Lotka-Volterra model the specific equations f_i are,

$$\frac{\mathrm{d}N_i}{\mathrm{d}t} = f_i(N_1, \ldots, N_S) = r_i N_i \left(1 - \sum_j^N \alpha_{ij} N_j \right) \ , \tag{2}$$

where r_i is the intrinsic growth rate of species i and α_{ij} is the interaction coefficient between species i and species j.

For a simple two-species model this can be written as,

$$\begin{cases} \dfrac{\mathrm{d}N_1}{\mathrm{d}t} = r_1 N_1 \left(1 - \alpha_{11} N_1 - \alpha 12 N_2\right) = r_1 N_1 - r_1 \alpha_{11} N_1^2 - r_1 \alpha_{12} N_1 N_2 \\[2ex] \dfrac{\mathrm{d}N_2}{\mathrm{d}t} = r_2 N_2 \left(1 - \alpha_{21} N_1 - \alpha 22 N_2\right) = r_2 N_2 - r_2 \alpha_{21} N_1 N_2 - r_2 \alpha_{22} N_2^2 \end{cases} \tag{3}$$

Then, any equilibrium point of the system is generally given by the solution of

$$\frac{\mathrm{d}N_i}{\mathrm{d}t} = f_i(N_1, \ldots, N_S) = 0 \ , \tag{4}$$

and is said to be feasible if all species have positive population densities.

*alex.gimenez@csic.es
†rob.salguero@biology.ox.ac.uk

In our specific simple two-species model, the equilibrium can be found by solving the following system of equations:

$$\begin{cases} 1 - \alpha_{11}N_1 - \alpha_{12}N_2 = 0 \\ \\ 1 - \alpha_{21}N_1 - \alpha_{22}N_2 = 0 \end{cases} . \tag{5}$$

From the first equation we can obtain an expression for N_1,

$$N_1 = -\frac{1 + \alpha_{12}N_2}{\alpha_{11}} , \tag{6}$$

which substituted into the second one gives rise to

$$N_2^* = \frac{\alpha_{21} - \alpha_{11}}{\alpha_{22}\alpha_{11} - \alpha_{21}\alpha_{12}} , \tag{7}$$

and substituting this last result into the previous expression for N_1 we obtain

$$N_1^* = -\frac{1 + \alpha_{12} \cdot \frac{\alpha_{21} - \alpha_{11}}{\alpha_{22}\alpha_{11} - \alpha_{21}\alpha_{12}}}{\alpha_{11}} . \tag{8}$$

The fixed point will be feasible if the values of the parameters α_{11}, α_{12}, α_{21} and α_{22} are such that N_1^* and N_2^* are positive. In other words, depending on these values the equilibrium will be feasible or not.

Let's assume we have a value of the parameters that conform to a feasible equilibrium point. Then, as explained in Box 1, the stability of the equilibrium point is given by the eigenvalues of the community matrix, which is the Jacobian matrix of the system evaluated at the equilibrium point,

$$\boldsymbol{M} = \boldsymbol{J}^* \quad \text{with} \quad m_{ij} = \frac{\partial f_i}{\partial N_j}\bigg|_{\boldsymbol{N}^*} . \tag{9}$$

We can easily compute the Jacobian matrix of our two-species Lotka-Volterra model,

$$\begin{cases} \dfrac{\partial f_1}{\partial N_1} = r_1 - 2r_1\alpha_{11}N_1 - r_1\alpha_{12}N_2 \\ \dfrac{\partial f_1}{\partial N_2} = -\alpha_{12}r_1N_1 \\ \dfrac{\partial f_2}{\partial N_1} = -r_2\alpha_{21}N_2 \\ \dfrac{\partial f_2}{\partial N_2} = r_2 - r_2\alpha_{21}N_1 - 2r_2\alpha_{22}N_2 \end{cases} , \tag{10}$$

so that the Jacobian matrix is

$$\boldsymbol{J} = \begin{pmatrix} \frac{\partial f_1}{\partial N_1} & \frac{\partial f_1}{\partial N_2} \\ \frac{\partial f_2}{\partial N_1} & \frac{\partial f_2}{\partial N_2} \end{pmatrix} = \begin{pmatrix} r_1 - 2r_1\alpha_{11}N_1 - r_1\alpha_{12}N_2 & -\alpha_{12}r_1N_1 \\ -r_2\alpha_{21}N_2 & r_2 - r_2\alpha_{21}N_1 - 2r_2\alpha_{22}N_2 \end{pmatrix} \tag{11}$$

To obtain the community matrix, $\boldsymbol{M}$, we just need to substitute N_1 and N_2 in the Jacobian for the corresponding equilibrium values.

2 Example of the derivation of the structured community matrix for a community with S species and 2 stages.

Consider a dynamical system representing a community of age/stage-structured populations with S species and K stages for each species. The dynamics of the system can be described by a general

system of ODEs that describes the growth rate of each population stage as a function of the abundances of all stages of all species (including the focal species) in the community,

$$\frac{\mathrm{d}N_i^{(k)}}{\mathrm{d}t} = g_i^{(k)}\left(N_1^{(1)},\dots,N_1^{(K)},\dots,N_S^{(1)},\dots,N_S^{(K)}\right) \ , \tag{1}$$

where $N_i^{(k)}$ denotes the density of the stage k of species i, with $i = 1, ..., S$ and $k = 1, ..., K$. This set of ODEs can incorporate any desired ecological mechanisms (e.g., transfers between stages due to reproduction or ageing, any explicit functional form of species interaction, etc).

In our simpler two-stage model, we can explicitly write an equation for each of the species stages. For simplicity, consider that the first stage is the juvenile state $k = 1 = J$ and the second one is the adult state, $k = 2 = A$, so that $N_i^{(k)}$ are J_i $(k = 1)$ and A_i $(k = 2)$,

$$\begin{cases} \frac{\mathrm{d}J_i}{\mathrm{d}t} = g_i^J\left(N_1^A, N_1^J,\dots,N_S^A,\dots,N_S^J\right) \\ \frac{\mathrm{d}A_i}{\mathrm{d}t} = g_i^A\left(N_1^A, N_1^J,\dots,N_S^A,\dots,N_S^J\right) \end{cases} , \tag{2}$$

To perform the change of variables we consider the total density of each species and the fraction of density in each stage,

$$N_i = \sum_{k=1}^{K} N_i^{(k)} \tag{3}$$

$$Z_i^{(k)} = \frac{N_i^{(k)}}{N_i} \quad \forall k = 1, ..., K-1 \ , \tag{4}$$

where N_i represents the total density of species i, $Z_i^{(k)}$ is the fraction of population density at stage k of species i. And we note that the fraction of population density at stage K is automatically defined by

$$Z_i^{(K)} = \left(1 - \sum_{k=1}^{K-1} Z_i^{(k)}\right) \tag{5}$$

In our simpler two-stage model, this is equivalent to the following equations,

$$N_i = J_i + A_i \tag{6}$$

$$Z_i^J = \frac{J_i}{N_i} \quad \forall k = 1, ..., K-1 \ , \tag{7}$$

where N_i represents the total density of species i, Z_i^J is the fraction of population density at the juvenile stage k of species i, and the fraction of population density at the adult stage (equivalent to stage K) is automatically defined by

$$Z_i^A = 1 - Z_i^J \tag{8}$$

We can then express our system of ODEs Eq. (1) as a function of total abundances Eq. (3) and fraction of stages Eq. (4),

$$\begin{aligned} \frac{\mathrm{d}N_i}{\mathrm{d}t} &= f_i\left(N_j, Z_j^{(1)},\dots,Z_j^{(K-1)}\right) \\ \frac{\mathrm{d}Z_i^{(k)}}{\mathrm{d}t} &= f_i^{(k)}\left(N_j, Z_j^{(1)},\dots,Z_j^{(K-1)}\right) \end{aligned} , \tag{9}$$

where $k \in 1, ..., K-1$ and $j \in 1, ..., S$.

For our simpler two-stage model, using Eqs. (6) and (7) this gets reduced to,

$$\begin{aligned} \frac{\mathrm{d}N_i}{\mathrm{d}t} &= f_i\left(N_j, Z_j^J\right) \\ \frac{\mathrm{d}Z_i^J}{\mathrm{d}t} &= f_i^J\left(N_j, Z_j^J\right) \end{aligned} , \tag{10}$$

Finally, we define the structured community matrix, $\boldsymbol{M}_S$ as the Jacobian of the age/stage structured population model, after the change of variables, evaluated at a feasible equilibrium point (denoted by *), which is a $SK \times SK$ matrix given by

$$
\boldsymbol{M}_S = \begin{pmatrix} \frac{\partial f_i}{\partial N_j} & \frac{\partial f_i}{\partial Z_j^{(1)}} & \cdots & \frac{\partial f_i}{\partial Z_j^{(K-1)}} \\ \frac{\partial f_i^{(1)}}{\partial N_j} & \frac{\partial f_i^{(1)}}{\partial Z_j^{(1)}} & \cdots & \frac{\partial f_i^{(1)}}{\partial Z_j^{(K-1)}} \\ \vdots & \vdots & \ddots & \vdots \\ \frac{\partial f_i^{(K-1)}}{\partial N_j} & \frac{\partial f_j^{(K-1)}}{\partial Z_j^{(1)}} & \cdots & \frac{\partial f_i^{(K-1)}}{\partial Z_j^{(K-1)}} \end{pmatrix}^* = \begin{pmatrix} \boldsymbol{M} & \boldsymbol{M}_S^{12} & \dots & \boldsymbol{M}_S^{1K} \\ \boldsymbol{M}_S^{21} & \boldsymbol{M}_S^{22} & \dots & \boldsymbol{M}_S^{2K} \\ \vdots & \vdots & \ddots & \vdots \\ \boldsymbol{M}_S^{K1} & \boldsymbol{M}_S^{K2} & \dots & \boldsymbol{M}_S^{KK} \end{pmatrix} \tag{11}
$$

in which each element of the depicted matrix is, in turn, a $S \times S$ matrix. Note that the sub-matrix $\boldsymbol{M}_S^{11}$ of the structured community matrix, $\left(\frac{\partial f_i}{\partial N_j}\right)^*$, is the usual community matrix, $\boldsymbol{M}$, representing the interactions between total species abundances. The remaining sub-matrices represent the interactions between the total species abundances and the fraction of stages (i.e., first row and first column of the matrix) and between the fraction of the different stages of the species.

We can write the structured community matrix in more detail in our simpler model,

$$
\boldsymbol{M}_S = \begin{pmatrix} \frac{\partial f_i}{\partial N_j} & \frac{\partial f_i}{\partial Z_j^J} \\ \frac{\partial f_i^{(J)}}{\partial N_j} & \frac{\partial f_i^J}{\partial Z_j^J} \end{pmatrix}^* = \begin{pmatrix} \begin{pmatrix} \frac{\partial f_1}{\partial N_1} & \frac{\partial f_1}{\partial N_2} & \cdots & \frac{\partial f_1}{\partial N_S} \\ \frac{\partial f_2}{\partial N_1} & \frac{\partial f_2}{\partial N_2} & \cdots & \frac{\partial f_2}{\partial N_S} \\ \vdots & \vdots & \ddots & \vdots \\ \frac{\partial f_S}{\partial N_1} & \frac{\partial f_S}{\partial N_2} & \cdots & \frac{\partial f_S}{\partial N_S} \end{pmatrix}^* & \begin{pmatrix} \frac{\partial f_1}{\partial Z_1^J} & \frac{\partial f_1}{\partial Z_2^J} & \cdots & \frac{\partial f_1}{\partial Z_S^J} \\ \frac{\partial f_2}{\partial Z_1^J} & \frac{\partial f_2}{\partial Z_2^J} & \cdots & \frac{\partial f_2}{\partial Z_S^J} \\ \vdots & \vdots & \ddots & \vdots \\ \frac{\partial f_S}{\partial Z_1^J} & \frac{\partial f_S}{\partial Z_2^J} & \cdots & \frac{\partial f_S}{\partial Z_S^J} \end{pmatrix}^* \\ \begin{pmatrix} \frac{\partial f_1^J}{\partial N_1} & \frac{\partial f_1^J}{\partial N_2} & \cdots & \frac{\partial f_1^J}{\partial N_S} \\ \frac{\partial f_2^J}{\partial N_1} & \frac{\partial f_2^J}{\partial N_2} & \cdots & \frac{\partial f_2^J}{\partial N_S} \\ \vdots & \vdots & \ddots & \vdots \\ \frac{\partial f_S^J}{\partial N_1} & \frac{\partial f_S^J}{\partial N_2} & \cdots & \frac{\partial f_S^J}{\partial N_S} \end{pmatrix}^* & \begin{pmatrix} \frac{\partial f_1^J}{\partial Z_1^J} & \frac{\partial f_1^J}{\partial Z_2^J} & \cdots & \frac{\partial f_1^J}{\partial Z_S^J} \\ \frac{\partial f_2^J}{\partial Z_1^J} & \frac{\partial f_2^J}{\partial Z_2^J} & \cdots & \frac{\partial f_2^J}{\partial Z_S^J} \\ \vdots & \vdots & \ddots & \vdots \\ \frac{\partial f_S^J}{\partial Z_1^J} & \frac{\partial f_S^J}{\partial Z_2^J} & \cdots & \frac{\partial f_S^J}{\partial Z_S^J} \end{pmatrix}^* \end{pmatrix} = \begin{pmatrix} \boldsymbol{M}_S^{NN} = \boldsymbol{M} & \boldsymbol{M}_S^{NJ} \\ \boldsymbol{M}_S^{JN} & \boldsymbol{M}_S^{JJ} \end{pmatrix}, \tag{12}
$$

where we can see that the first sub-matrix of the structured community matrix is nothing but the community matrix, measuring the effect of a change in the abundance of species j on the growth rate of species i.

3 Homogeneous interaction types

We begin by examining communities where all interactions—adult-adult, adult-juvenile, juvenile-adult, and juvenile-juvenile—are of the same type. These homogeneous configurations include four scenarios: all random, all mutualistic (+/+), all competitive (-/-), or all predator-prey interactions.

We parametrise the structured community matrix (SCM) considering the following criteria:

- If $i \neq j$
  - **Random interactions**:
    - $M_{ij}^1 \sim \mathcal{N}(0, \sigma_1)$ with probability C, 0 otherwise.
    - $M_{ij}^2 \sim \mathcal{N}(0, \sigma_2)$ with probability C, 0 otherwise.
    - $M_{ij}^3 \sim \mathcal{N}(0, \sigma_3)$ with probability C, 0 otherwise.
    - $M_{ij}^4 \sim \mathcal{N}(0, \sigma_4)$ with probability C, 0 otherwise.

    With $\gamma_i = \sigma_i \sqrt{SC}$.

- **Mutualistic interactions**
  * $M^1_{ij} \sim |\mathcal{N}(0,\sigma_1)|$ with probability C, 0 otherwise.
  * $M^2_{ij} \sim |\mathcal{N}(0,\sigma_2)|$ with probability C, 0 otherwise.
  * $M^3_{ij} \sim |\mathcal{N}(0,\sigma_3)|$ with probability C, 0 otherwise.
  * $M^4_{ij} \sim |\mathcal{N}(0,\sigma_4)|$ with probability C, 0 otherwise.

  With $\gamma_i = \sigma_i C(S-1)\sqrt{\frac{2}{\pi}}$.
- **Competitive interactions**
  * $M^1_{ij} \sim -|\mathcal{N}(0,\sigma_1)|$ with probability C, 0 otherwise.
  * $M^2_{ij} \sim -|\mathcal{N}(0,\sigma_2)|$ with probability C, 0 otherwise.
  * $M^3_{ij} \sim -|\mathcal{N}(0,\sigma_3)|$ with probability C, 0 otherwise.
  * $M^4_{ij} \sim -|\mathcal{N}(0,\sigma_4)|$ with probability C, 0 otherwise.

  With $\gamma_i = \sigma_i \left\{\sqrt{SC}\left(1+\frac{2-2C}{\pi-2C}\right)\sqrt{\frac{\pi-2C}{\pi}} + C\sqrt{\frac{2}{\pi}}\right\}$.
- **Predator-prey interactions**

  With probability C each pair of species interact and with probability 1/2 species i predates on j (otherwise j predates on i).

  If species i predates on species j we consider (otherwise the sign relation is inverted):
  * $M^1_{ij} \sim |\mathcal{N}(0,\sigma_1)|$ and $M^1_{ji} = -|\mathcal{N}(0,\sigma_1)|$
  * $M^2_{ij} \sim |\mathcal{N}(0,\sigma_2)|$ and $M^2_{ji} = -|\mathcal{N}(0,\sigma_2)|$
  * $M^3_{ij} \sim |\mathcal{N}(0,\sigma_3)|$ and $M^3_{ji} = -|\mathcal{N}(0,\sigma_3)|$
  * $M^4_{ij} \sim |\mathcal{N}(0,\sigma_4)|$ and $M^4_{ji} = -|\mathcal{N}(0,\sigma_4)|$

  With $\gamma_i = \frac{\pi-2}{\pi}\sigma_i\sqrt{SC}$.

- If $i = j$
  - $M^1_{ii} = -d$
  - $M^2_{ii} \sim \mathcal{N}(0,\sigma_2)$
  - $M^3_{ii} \sim \mathcal{N}(0,\sigma_3)$
  - $M^4_{ii} = -d$

Recall that these matrices represent:

- M^1_{ij}: change in growth rate of the total abundance of species i after a change in total abundance of species j
- M^2_{ij}: change in growth rate of the fraction of juveniles in species i after a change in total abundance of species j
- M^3_{ij}: change in growth rate of the total abundance of species i after a change in the abundance of juveniles of species j
- M^4_{ij}: change in growth rate of the fraction of species i after a change in the abundance of the fraction of juveniles of species j

In all our simulations, we used $C = 1$, $\mu = 1$ and $S = 1000$. We used several values of γ (note that γ is a function of σ so that knowing the value of one completely determines the other) as can be observed in the panels of Fig. 1.

In these cases, communities predicted to be unstable by unstructured models remain unstable even when stage structure is introduced. However, if the strength of adult-juvenile (or juvenile-adult) interactions becomes sufficiently large, communities initially predicted to be stable by unstructured models may become unstable Fig. 1(A-F).

Following from the general result of Robert May [1], it would be expected that the volume of the phase space comprising stable communities would decrease if the interaction strength of total populations (γ_1) were to increase. This is observed in the case of random (not shown), mutualistic and predator-prey interactions, but not for competitive ones. We observe an intermediate point at higher values of total population to total population interaction strength in which the number of possible stable communities is maximised Fig. 1(G).

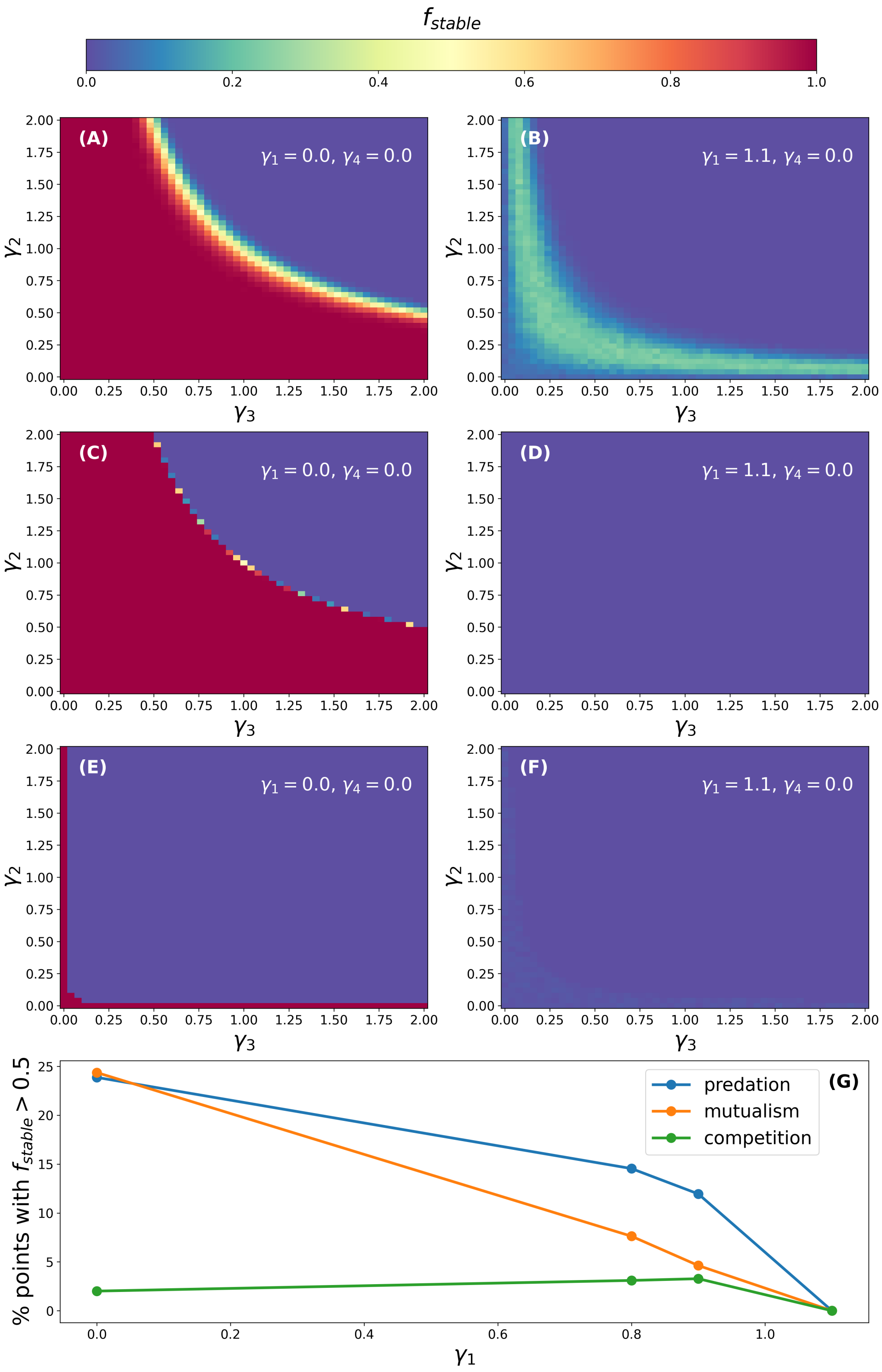

Figure 1: Stability analysis of communities with homogeneous random interactions. The proportion stable communities are shown for a case where all interaction types (adult-adult, adult-juvenile, juvenile-adult, and juvenile-juvenile) are (A-B) predator prey, (C-D) mutualistic, and (E-F) competitive. (G) Percentage of parameter combinations ((γ_2, γ_3), with respect of the total number of combinations explored) for which more than 50% of the simulated communities are stable.

4 Stage-asymmetric interaction types

For these scenarios, adult-adult and juvenile-juvenile interactions are of the same type but differ from the adult-juvenile and juvenile-adult interaction type. We switch off juvenile-juvenile interactions for simplicity.

The parametrisation of the SCM follows the same rules as before, but now each matrix (M^1 to M^4) will display a different sign relation depending on the interaction type considered between stages. For instance, the parametrisation of the SCM for a community with **adult-adult competition and adult-juvenile (and juvenile-adult) predator-prey interactions** would consist of the following rules:

- If $i \neq j$:
  - $M^1_{ij} \sim -|\mathcal{N}(0, \sigma_1)|$
  - $M^2_{ij} \sim |\mathcal{N}(0, \sigma_2)|$ and $M^2_{ji} = -|\mathcal{N}(0, \sigma_2)|$ (if species i predates on species j, otherwise signs inverted)
  - $M^3_{ij} \sim |\mathcal{N}(0, \sigma_3)|$ and $M^3_{ji} = -|\mathcal{N}(0, \sigma_3)|$ (if species i predates on species j, otherwise signs inverted)
  - $M^4_{ij} = 0$ (juvenile-juvenile interactions are not considered)
- If $i = j$:
  - $M^1_{ii} = -d$
  - $M^2_{ii} \sim \mathcal{N}(0, \sigma_2)$
  - $M^3_{ii} \sim \mathcal{N}(0, \sigma_3)$
  - $M^4_{ii} = -d$

Again, we consider $S = 1000$, $C = 1$ and $\mu = 1$ in all the simulations.

Adult-juvenile predator-prey interactions provide a stabilizing effect: communities predicted as **unstable** by unstructured population models can become **stable** when stage-structure is considered. This is independent of the adult-adult interaction type and occurs for intermediate values of the adult-juvenile interaction strength. For too low or too high values, the community is not stabilized. See Fig. 2. The effect is independent on the value of the pair-wise interaction probability, C (Fig. 3), and the distribution used to obtain the values of the interaction strengths (Fig. 4).

Competitive adult-juvenile interactions are destabilizing: communities predicted as **stable** by unstructured population models can become **unstable** when stage-structure is considered. This is independent of the adult-adult interaction type. See Fig. 5.

Mutualistic adult-juvenile interactions have a neutral effect: communities that would be predicted to be **unstable** by unstructured population models remain **unstable** when stage-structure is considered. Of course, high values of adult-juvenile interaction strength can also destabilize a community, as previously shown. See Fig. 6.

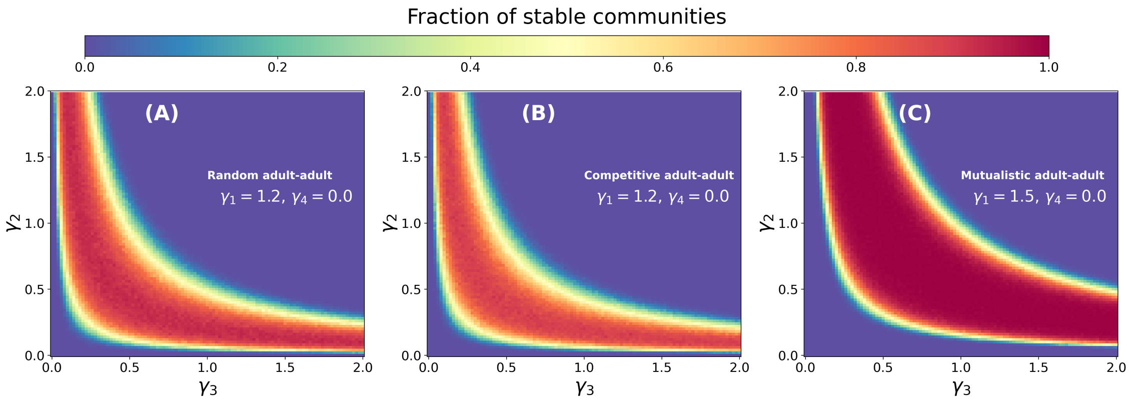

Figure 2: Stability analysis of communities with adult-juvenile predator-prey interactions. In all cases an unstructured population model would predict an unstable community ($\gamma_1 > 1$). We observe that intermediate values of adult-juvenile (γ_2) and juvenile-adult (γ_3) interaction strength promote community stability.

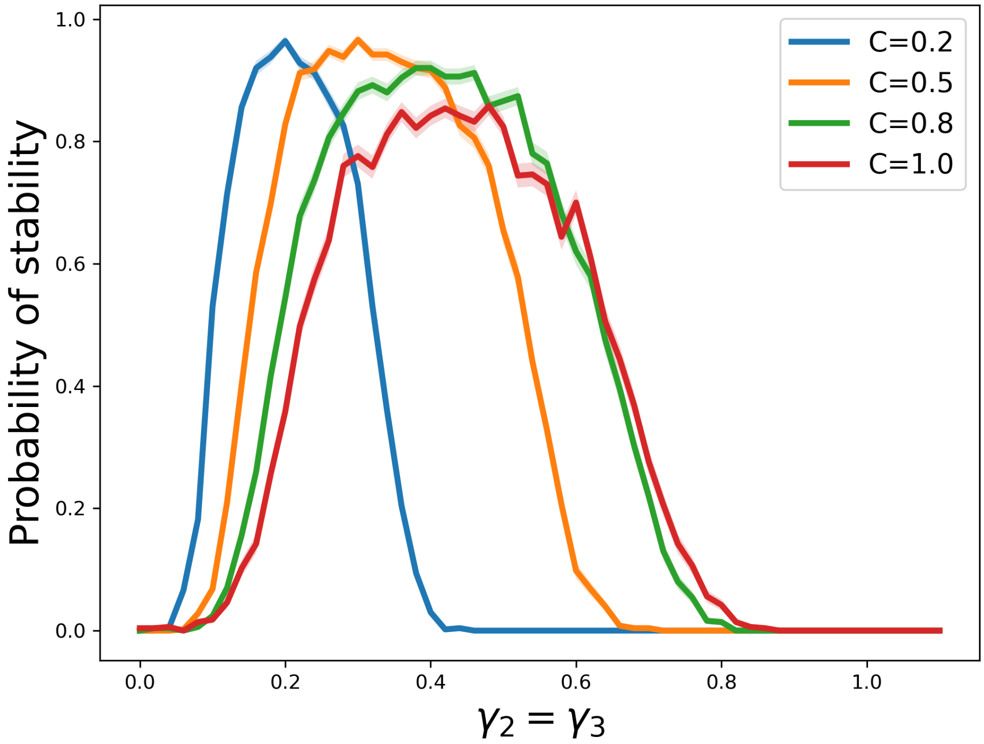

Figure 3: Stabilizing effect of cross-stage predation for different values of the pairwise interaction probability, C. The results correspond to the average proportion of stable communities in an ensemble of 500 realizations. The dashed area corresponds to the standard error.

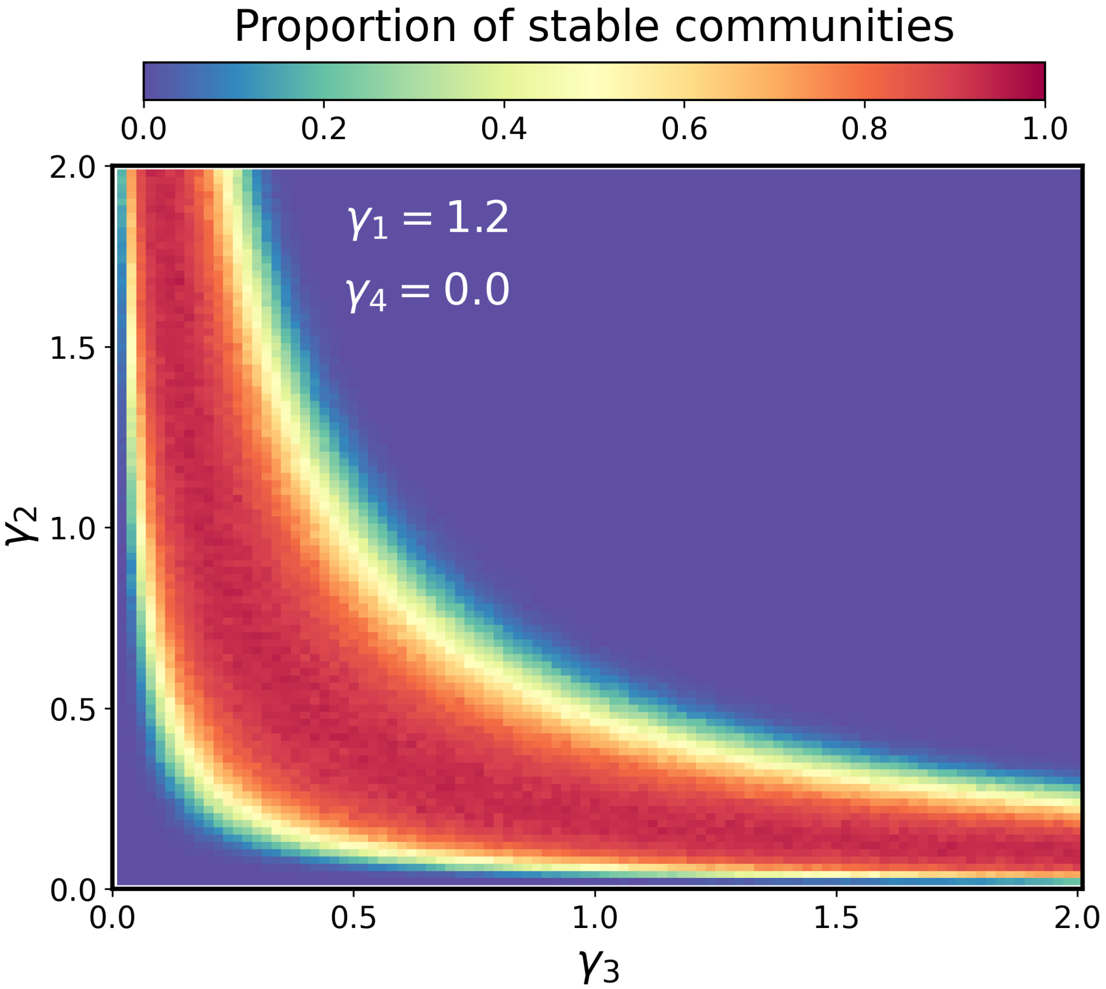

Figure 4: Effect of using a truncated normal distribution for interaction strengths. Proportion of stable communities as a function of the cross-stage interaction strengths γ_2 and γ_3, obtained from $N = 1000$ simulations where interaction coefficients were drawn from a truncated normal distribution (instead of a folded normal distribution). Parameters: $\gamma_1 = 1.2$, $\gamma_4 = 0$, $S = 200$, $C = 1$. The qualitative stability patterns remain unchanged, confirming that the results are robust to the specific form of the interaction strength distribution.

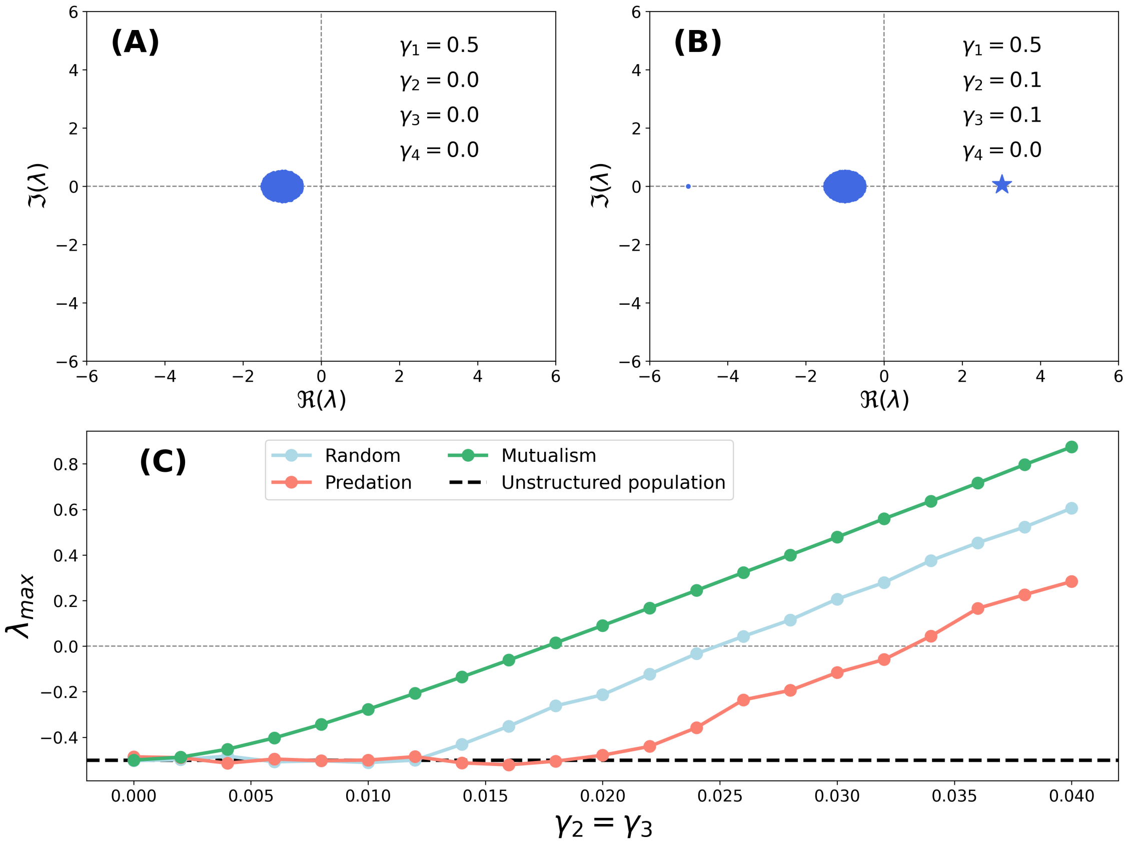

Figure 5: Stability analysis of communities with adult-juvenile competitive interactions. (A) Eigenvalues of the structured community matrix with $\gamma_1 = 0.5$ and $\gamma_2 = \gamma_3 = \gamma_4 = 0.0$, so that essentially the adult-juvenile and juvenile-juvenile interactions are switched off. (B) Eigenvalues of the structured community matrix with $\gamma_1 = 0.5$, $\gamma_2 = \gamma_3 = 0.1$ and γ_4, so that adult-juvenile interactions are switched on with quite low interaction strength and juvenile-juvenile interactions are still deactivated. We observe that $\text{Re}\{\lambda_{max}\} > 0$, making the community unstable. (C) This behaviour is observed independently on the adult-adult interaction type, although the transition to instability occurs at different values of the juvenile-adult (and adult-juvenile) interaction strength, $\gamma_2 = \gamma_3$. In this case, an structured population model would predict that the community is stable, so that competitive adult-juvenile interactions are destabilizing.

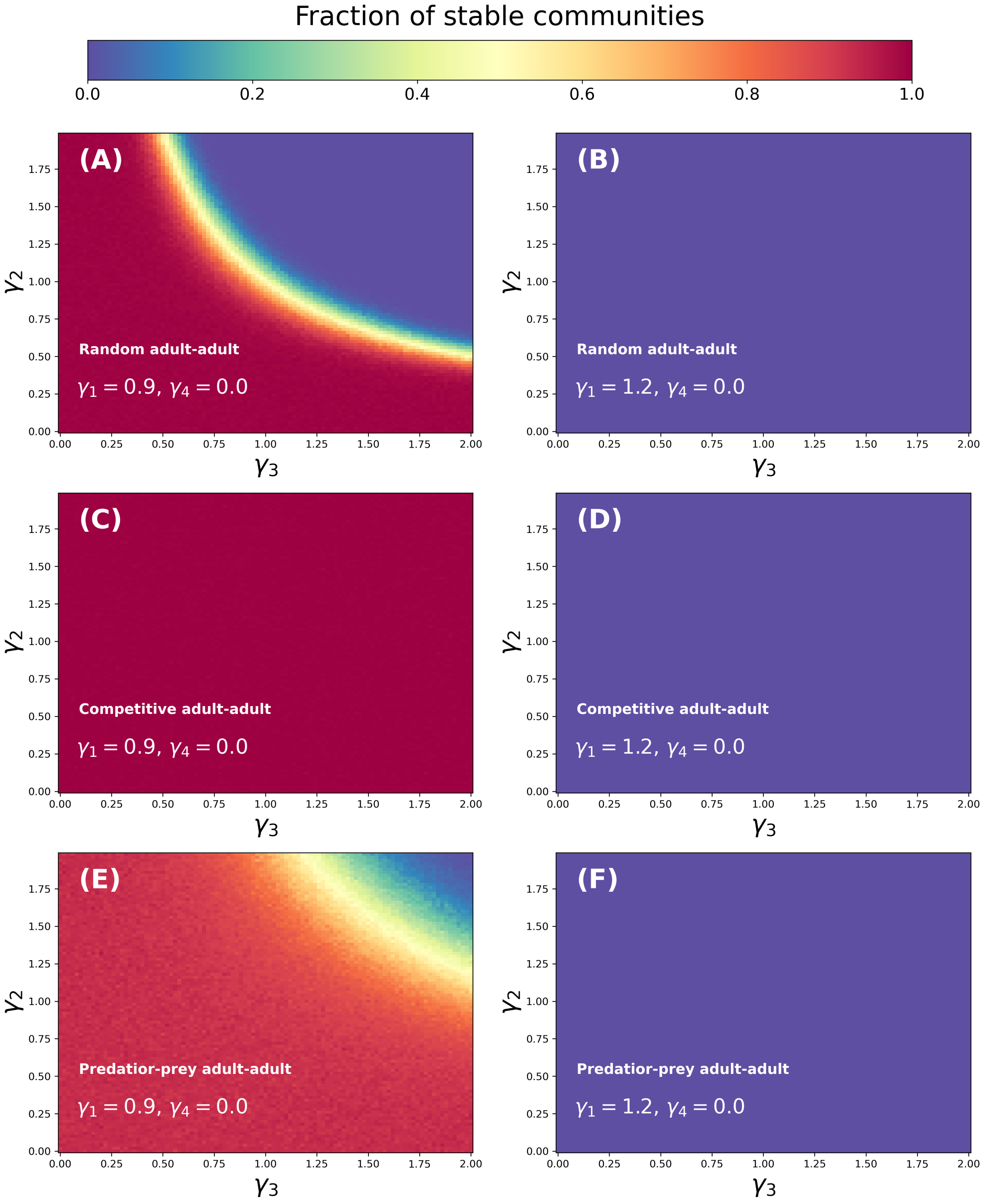

Figure 6: Stability analysis of communities with adult-juvenile mutualistic interactions. Each row corresponds to one type of adult-adult interaction: Random (A-B), competitive (C-D) and predator-prey (E-F). The first column corresponds to a case in which an unstructured population model would predict a stable community, while in the second it would predict an unstable one. Independently on the type of adult-adult interactions, mutualistic adult-juvenile interactions are not able to stabilise communities. On the other hand, if the adult-juvenile interaction strength is big enough, stable communities as predicted by unstructured population models can become unstable.

5 Example with empirical food webs

Here we apply our framework to study the stability of empirical food webs with the assumption that the predator-prey interactions occur between different stages of the species. Unfortunately, there is no empirical data in which the interactions among species are documented at a lower organizational level than that of species. Nevertheless, we can use available empirical food webs to constrain the interaction network at the species level (e.g. fixing the number of species, S, and the connectivity, C, from empirical data) and then consider a specific form of the intra- and inter-stage interactions (e.g. the adults of the interacting species compete while adults predate the juveniles).

We obtained 33 food web networks from Web of Life, which constrain the number of species, S, connectivity, C, and many other properties of the interaction networks empirically. The species richness of the analysed food-webs span from $S = 14$ to $S = 249$ and connectivity values range from $C = 0.035$ to $C = 0.485$ (Fig. 7) .

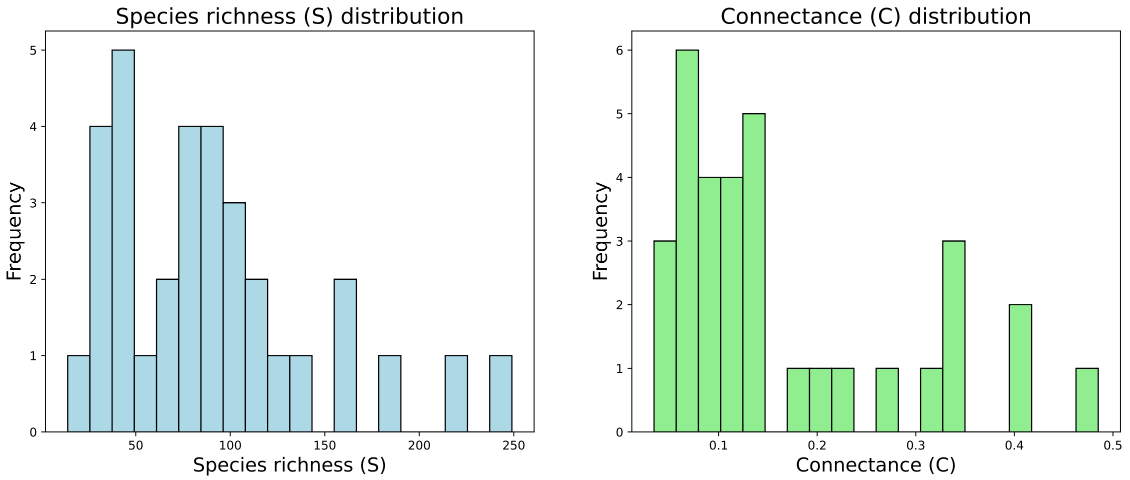

Figure 7: Distribution of species richness, S, and connectivity, C, for the 33 analysed food-webs from web of life.

We then considered that each of the species is organized into two different stages (i.e. adults and juveniles), and that the predator-prey interaction occurs between the adults and the juveniles of the interacting species while the adults and juveniles have random interactions (i.e., can have any interaction type). Again, for the sake of simplicity, we will set the juvenile-juvenile interactions to zero for the analysis. Thus, we are basically testing our theoretical insights with adult-juvenile predator-prey interactions with empirical food web networks.

The parametrisation of the SCM follows the same rules as those of the previous section. In particular, the parametrisation of the SCM for a community with **adult-adult random interactions** and **adult-juvenile predator-prey interactions** would consist of the following rules:

- If $i \neq j$:
  - $M^1_{ij} \sim \mathcal{N}(0, \sigma_1)$
  - $M^2_{ij} \sim |\mathcal{N}(0, \sigma_2)|$ and $M^2_{ji} = -|\mathcal{N}(0, \sigma_2)|$ (if species i predates on species j, otherwise signs inverted)
  - $M^3_{ij} \sim |\mathcal{N}(0, \sigma_3)|$ and $M^3_{ji} = -|\mathcal{N}(0, \sigma_3)|$ (if species i predates on species j, otherwise signs inverted)
  - $M^4_{ij} = 0$ (juvenile-juvenile interactions are not considered)
- If $i = j$:
  - $M^1_{ii} = -d$

- $M^2_{ii} \sim \mathcal{N}(0, \sigma_2)$
- $M^3_{ii} \sim \mathcal{N}(0, \sigma_3)$
- $M^4_{ii} = -d$

As in our theoretical example, we set the intra-specific regulation to $d = 1$ and the total-population to total-population interaction strength, $\gamma_1 = 1.2(> d)$ greater than the self-regulation term. This means that the corresponding unstructured population model would be unstable (i.e. the largest eigenvalue of the community matrix is positive, $\lambda_1^M > 0$).

As shown in Figs. 8 and 9 most of the empirical food webs are stabilized by predator-prey cross-stage interactions for intermediate values of the cross-stage interaction strength, γ_2 and γ_3. In **??**, we show the average probability of stability by taking into account the 33 empirical food webs.

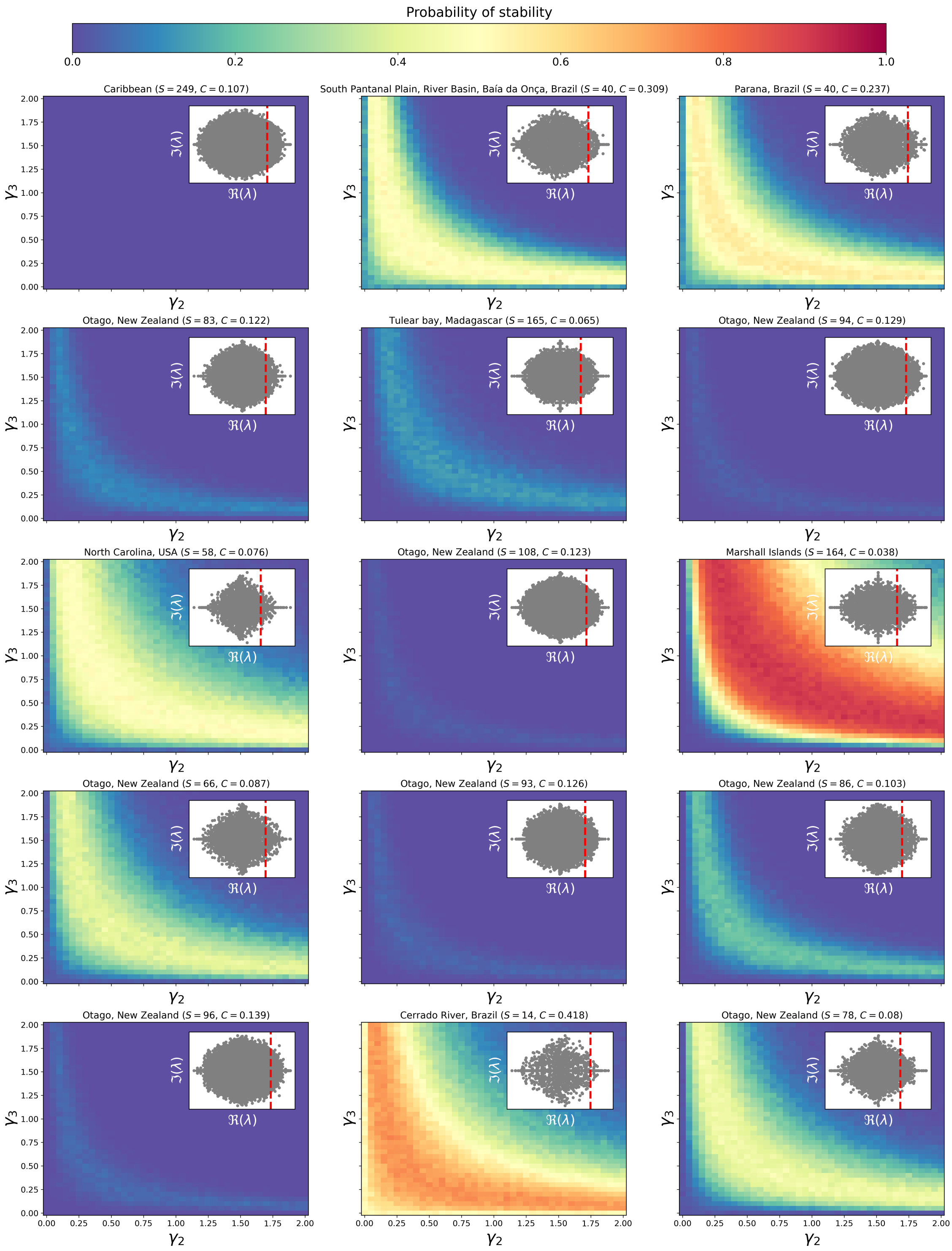

Figure 8: Stability analysis of empirical food webs. Each panel correspond to a different community obtained from web of life, for which the location of the community and the number of species, S, and connectivity, C, of the interaction network is specified on top of the panel. The main plot shows the probability of stability for different values of the cross-stage interaction strength, γ_2 and γ_3, while $\gamma_1 = 1.2$ and $\gamma_4 = 0$ in all cases. The inset shows the eigenvalue distribution of the corresponding unstructured population model, which is always unstable as $\gamma_1 > d = 1$.

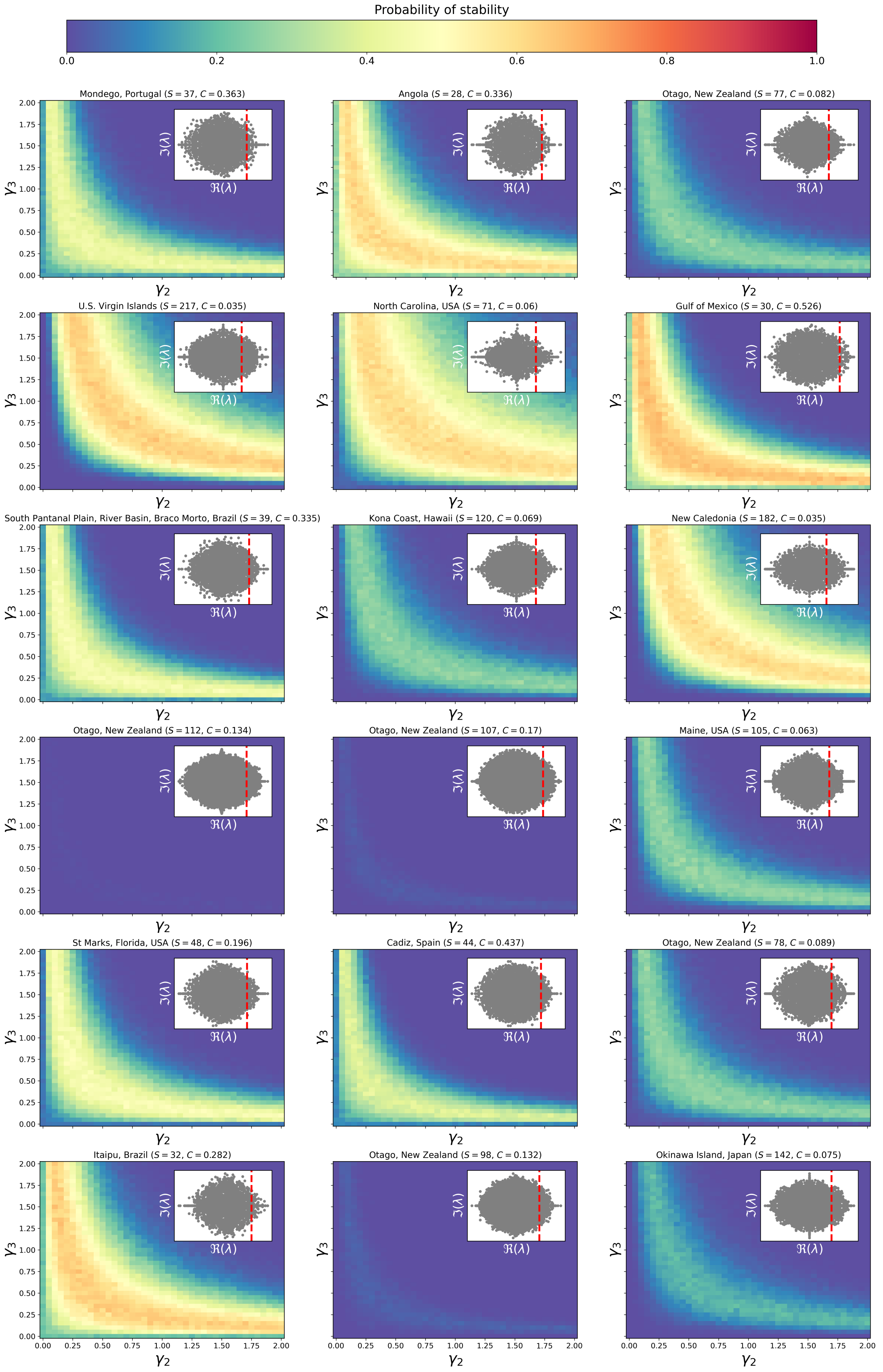

Figure 9: Stability analysis of empirical food webs. Same as Fig. 8 for different food webs.

References

[1] R. M. May, "Will a Large Complex System be Stable?", Nature **238**, 413–414 (1972).
