# Supplementary figures and images for: Population Structure Plays a Key Role in Community Stability
Source: Ecol Lett. 2025 Dec 8;28(12):e70272. doi: 10.1111/ele.70272 (PMC12685569; doi:10.1111/ele.70272)

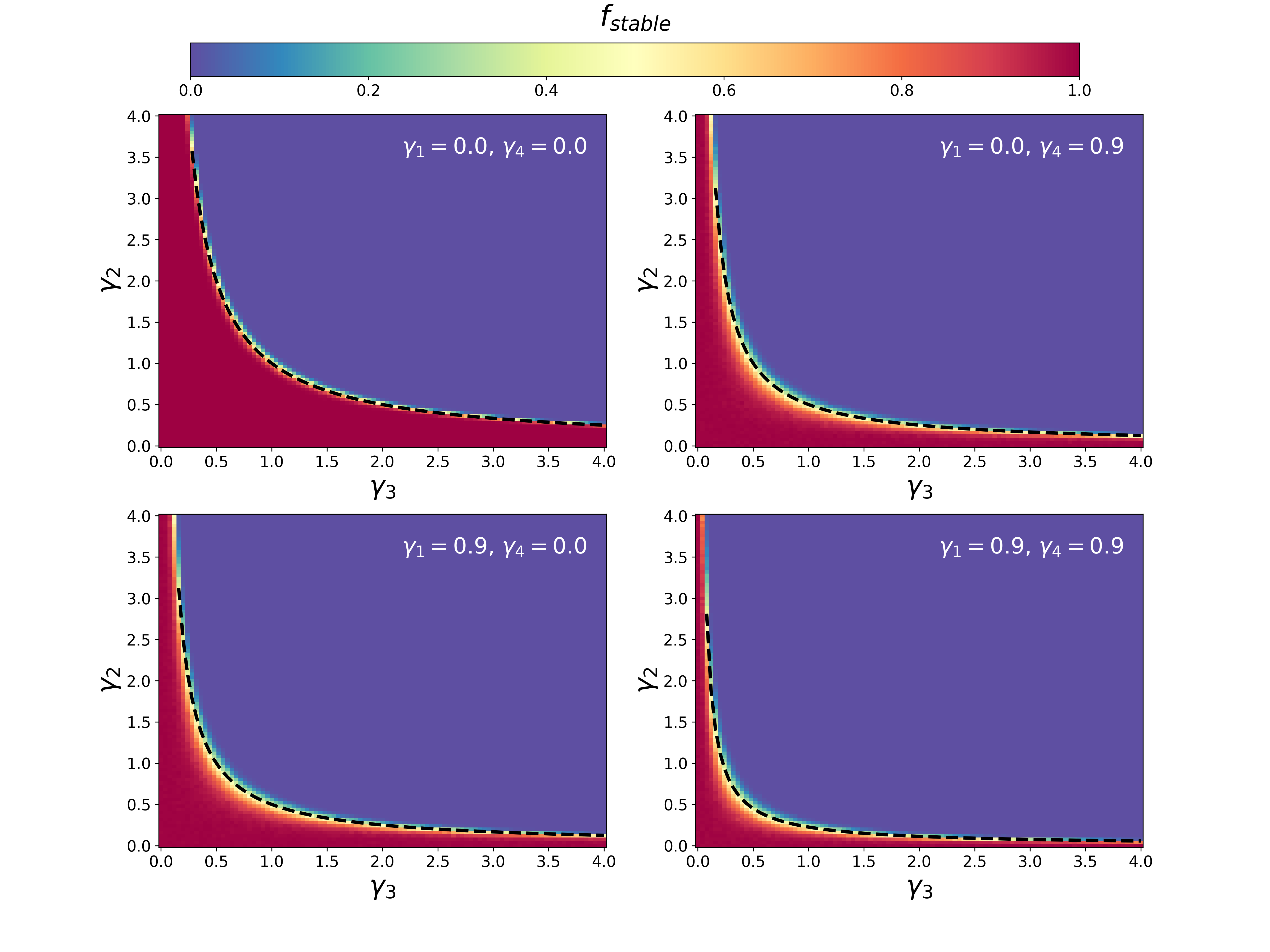

Supplement: Supplementary file 2 — Data S1: ele70272‐sup‐0002‐DataS1.png. [file ELE-28-0-s002.png]
